# Edible plant oils modulate gut microbiota during their health-promoting effects: a review

**DOI:** 10.3389/fnut.2024.1473648

**Published:** 2024-09-30

**Authors:** Qi Zou, Ao-Qiu Chen, Jing Huang, Mei Wang, Jiang-Hong Luo, An Wang, Xiao-Yin Wang

**Affiliations:** ^1^Key Laboratory of Prevention and Treatment of Cardiovascular and Cerebrovascular Diseases, Ministry of Education, Gannan Medical University, Ganzhou, China; ^2^School of Public Health and Health Management, Gannan Medical University, Ganzhou, China; ^3^State Center of Quality Testing and Inspection for Camellia Products, Ganzhou, China; ^4^Key Laboratory of Development and Utilization of Gannan Characteristic Food Function Component of Ganzhou, Gannan Medical University, Ganzhou, China

**Keywords:** edible plant oils, gut microbiota, health-promoting effects, metabolites, correlations, nutritional foods

## Abstract

Edible plant oils are widely used in cooking, cosmetics, health supplement capsules, and other industries, due to their various health-promoting effects. There is increasing evidence that edible plant oils can modulate gut microbiota during their health-promoting effects in animal experiments and cohort or clinical studies. However, the information concerning the gut microbiota modulation of edible plant oils during their health-promoting effects is scattered. In this article, the research progress on gut microbiota modulation of edible plant oils (especially camellia oil, olive oil, and flaxseed oil) is summarized. Meanwhile, a summary on correlations between modulated gut microbiota and changed biochemical indexes is provided. The alterations of edible plant oils on gut microbiota-derived metabolites and the correlations between altered metabolites and modulated gut microbiota as well as changed biochemical indexes are reviewed. Furthermore, the prospects for gut microbiota modulation of edible plant oils during their health-promoting effects are put forward. Existing literature has shown that edible plant oils could modulate gut microbiota during their health-promoting effects, and some differential gut microbiota biomarkers were gained. Some similarities and differences existed while the oils exhibited health-promoting actions. Dosage and treatment time have influences on gut microbiota modulation of edible plant oils. Different edible plant oils exhibited different behaviors in modulating gut microbiota, and edible plant oils were mostly different in modulating gut microbiota compared to edible animal oils. Moreover, the modulated gut microbiota was significantly correlated with the changed biochemical indexes. Furthermore, edible plant oils altered SCFAs and other gut microbiota-derived metabolites. The altered metabolites were obviously correlated with the modulated gut microbiota and changed biochemical indexes. This review is helpful to the future research and application of edible plant oils in health-promoting effects from the perspective of gut microbiota.

## 1 Introduction

Edible plant oils, obtained from the seeds, pulps, fruits, or plumules of certain plants, are an important source of dietary fat and represent as much as 25% of human caloric intake in developed countries (1). They are widely used in cooking, food, pharmaceutical, cosmetics, and other industries (2). With the increase in the world population, the demand for edible oils is increasing, showing the annual growth rate of global demand for edible plant oils as 5.14% from 2020 to 2025 (3). In 2022, the global consumption of edible plant oils was 212.82 million tons, showing a 1.25-fold increase compared to 2014 (4). A more important reason behind this was the health-promoting effects of them, due to their special fatty acid composition and abundant bioactive components.

Compared with most animal oils, edible plant oils are rich in unsaturated fatty acids and lacking cholesterol. Moreover, there are a diversity of bioactive compounds in edible plant oils, such as sterol, squalene, polyphenols, and tocopherols. As research continues, more attention has been increasingly paid to the edible plant oils’ health-promoting effects, including antioxidant (5), anti-inflammatory (6), regulation of lipid metabolism (7), anti-Alzheimer’s disease (8), and anti-cancer (9) activities, as well as other activities. For example, camellia oil has been reported to possess antioxidant, anti-cancer, anti-inflammatory, antibacterial, antihypertensive, hypoglycemic, cardioprotective, and immunoregulatory activities (10). Olive oil has been reviewed to have anti-inflammatory, chemopreventive, antimicrobial, hepatoprotective, kidney protection, and anti-neurodegenerative effects (11). Flaxseed oil has been summarized to have antioxidant, anti-inflammatory, anti-obesity, and bone osteoporosis improvement actions (12). Coconut oil has been reported to possess hypocholesterolemic, anti-cancer, antihepatosteatotic, antidiabetic, antioxidant, anti-inflammatory, antimicrobial, and skin moisturizing properties (13).

The trillions of microorganisms in the human intestine are important regulators of health (14). Gut microbiota is considered as an environmental factor that interacts with diet and may also have an impact on health outcomes, many of which involve metabolites produced by the microbiota from dietary components that can impact the host (15). In recent years, a lot of edible plant oils have been demonstrated to modulate gut microbiota during their health-promoting effects in numerous animal experiments (16–18) and cohort or clinical studies (19–21). For instance, camellia oil has been demonstrated to exhibit anti-fatigue properties by modulating the gut microbial composition of mice, which were received with Rotarod test and Treadmill test (16). Olive oil-enriched diet has been indicated to increase the abundance of lactic acid bacteria in overweight/obese subjects (19). Perilla oil has been found to relieve constipation and enhance diversity of gut microbiota in sedentary healthy female (21). Among them, camellia oil, olive oil, and flaxseed oil are three of the common edible plant oils on the market. Especially, these three oils have been extensively reported to modulate gut microbiota during their health-promoting effects.

Currently, the research progress on health-promoting effects of edible plant oils has been summarized in some literature (10–12, 22). Moreover, there were several reviews that partly summarized some findings about the modulations of edible plant oils (such as olive oil, flaxseed oil, safflower oil, and palm oil) on gut microbiota during their health-promoting effects (23–25). However, the information concerning the gut microbiota modulation of edible plant oils during their health-promoting effects is still scattered. It is necessary to conduct a comprehensive review on this aspect for better understanding of the health-promoting effects of edible plant oils from the perspective of gut microbiota.

Herein, the research progress on gut microbiota modulation of edible plant oils (especially camellia oil, olive oil, and flaxseed oil) is reviewed. Meanwhile, the correlations between modulated gut microbiota and changed biochemical indexes are summarized. The alterations of edible plant oils on gut microbiota-derived metabolites and the correlations between altered metabolites and modulated gut microbiota as well as changed biochemical indexes are reviewed. Furthermore, the prospects for gut microbiota modulation of edible plant oils in exhibiting health-promoting effects are put forward.

## 2 Overview of chemical composition in edible plant oils

Camellia oil is extracted from the seeds of *Camellia oleifera* Abel. The unsaturated fatty acid content of camellia oil is as high as 85–97%, consisting mainly of oleic (71.42–90%) and linoleic (7–14%) acids. And the saturated fatty acid content is usually approximately 10–13%, consisting mainly of palmitic acid (7–9%), stearic acid (1–3%), and a small amount of palmitoleic acid (26). Meanwhile, it also contains a multitude of bioactive components, such as sterol (2860.18–4748.39 mg/kg), squalene (122.02–248.24 mg/kg), polyphenols (20.56–88.56 mg/kg), sasanquasaponin (38.5 mg/kg), tocopherols (*α*-tocopherol, 153–771 mg/kg; *γ*-tocopherol, 9.4–59 mg/kg; *δ*-tocopherol, 0.27–28 mg/kg) and other functional substances (26, 27).

Olive oil is obtained from the fruit of *Olea europaea* L. It is composed of ~98–99% of fatty acids, mainly triacylglycerol esters of oleic acid (55–83%), palmitic acid (7.5–20%), linoleic acid (3.5–21%), and other fatty acids such as stearic acid (0.5–5%) (28). The unsaponifiable fraction of olive oil includes triterpenic dialcohols and acids (20–200 mg/kg), sterols (1000–5,000 mg/kg), squalene (1,000–8,000 mg/kg), pigments (5–30 mg/kg), and phenolic compounds (50–1,000 mg/kg) (9, 29).

Flaxseed oil is gained from the seeds of *Linum usitatissimum* L. It has high unsaturated fatty acids (>70%), mainly composed of linolenic acid (53.36–65.84%), linoleic acid (10.14–16.39%), oleic acid (10.03–12.37%), stearic acid (3.98–9.85%), and palmitic acid (2.41–7.97%) (12, 30). In addition, it contains many bioactive compounds, including sterols (0.25–0.3%), polyphenols (15.69–47.68 mg/kg), cyclic polypeptides (188.6–643.8 mg/kg), tocopherol (374–563.7 mg/kg), lignans (0–32, 28 mg/kg), chlorophyll (1.45–2.08 mg/kg), carotenoid (2.89–3.45 mg/kg), and other compounds (12).

Other edible plant oils are also rich in unsaturated fatty acids, mainly composed of oleic acid (soybean oil, 15–36%; peony seed oil, 20.5–45.1%; walnut oil, 10–20%; etc.), linoleic acids (soybean oil, 42.8–56.1%; peony seed oil, 16.5–33.6%; walnut oil, 55–70%; etc.) and linolenic acids (soybean oil, 2–14%; peony seed oil, 28.1–46.9%; walnut oil, 10–18%; etc.) (2). Meanwhile, other edible plant oils contain many bioactive ingredients, including phytosterols (safflower oil, 243.7 mg/100 g; peony seed oil, 154.5 mg/100 g; walnut oil, 115.7 mg/100 g; etc.), phenolic compounds (safflower oil, 231.40 mg/kg; *Torreya grandis* seed oil, 12,630 mg/kg; almond oil, 644.54 mg/kg; etc.), tocopherol (sea buckthorn seed oil, 898.1 mg/kg; tomato seed oil, 345.8 mg/kg; rice bran oil, 322.7 mg/kg; etc.), squalene (walnut oil, 12 mg/100 g; peony seed oil, 4 mg/100 g; rice bran oil, 24 mg/100 g; etc.), and *β*-carotene (tomato seed oil, 765.7 mg/kg; sea buckthorn seed oil, 55.3 mg/kg; corn oil, 0.07 mg/kg; etc.) (22).

## 3 Gut microbiota modulation of edible plant oils

### 3.1 Camellia oil modulation

**Figure 1:**
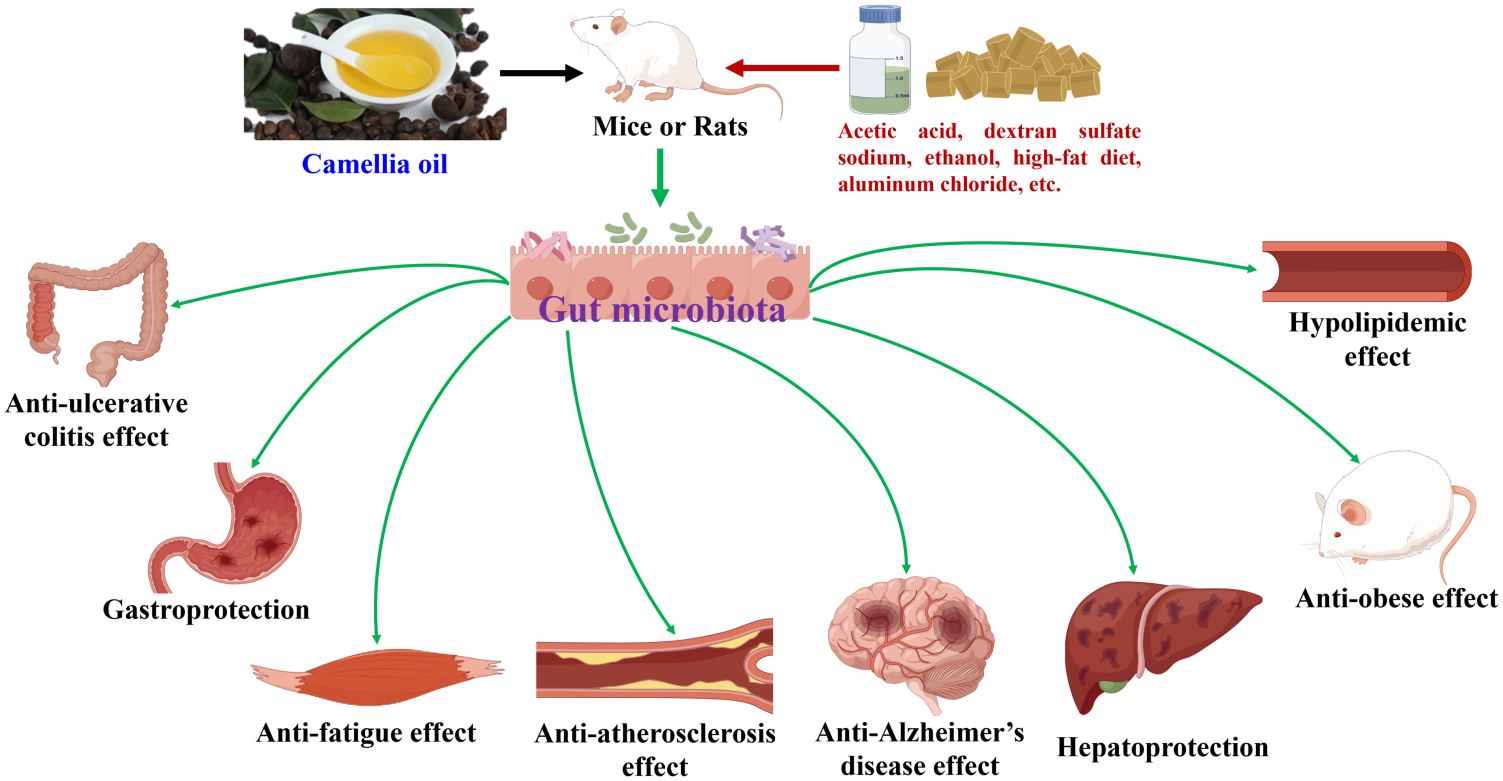
Camellia oil modulates gut microbiota during its health-promoting effects.

Camellia oil has been demonstrated to modulate gut microbiota during its health-promoting effects (8, 16, 31–42), as shown in Table 1 and Figure 1. In terms of anti-ulcerative colitis effect, supplementation of camellia oil increased the abundances of Firmicutes and/or Firmicutes/Bacteroidetes ratio, and/or decreased that of Bacteroidetes in acetic acid and dextran sulfate sodium-induced rats or mice, at phylum level (32, 33). And, at the genus level, camellia oil upregulated the amounts of *Bacteroides* and *Lactobacillus* and downregulated those of *Alistipes* and *Lachnospiraceae NK4A136 group* in dextran sulfate sodium-induced colitis mice (33). Moreover, Actinobacteria, Bacteroidetes, Firmicutes, and Proteobacteria were differential gut microbiota biomarkers for camellia oil in anti-ulcerative colitis against acetic acid-induced colitis rats (32).

**Table 1:** Gut microbiota modulation of camellia oil during its health-promoting effects.

Health-promoting effect	Experimental model	Oil dosage and treatment time	Gut microbiota modulation	Gut microbiota biomarker	References
Anti-ulcerative colitis	Acetic acid-induced rats	2 mL/kg BW, 3 weeks	*Lactobacillus* spp. and *Bifidobacterium* spp. (+)	NA	(31)
Anti-ulcerative colitis	Acetic acid-induced rats	2 mL/kg BW, 20 d	Phylum level: Firmicutes (+); Bacteroidetes (−)	Actinobacteria, Bacteroidetes, Firmicutes and Proteobacteria	(32)
Anti-ulcerative colitis	Dextran sulfate sodium-induced mice	0.5, 1 and 2 mL/kg BW, 3 weeks	Phylum level: Firmicutes and Firmicutes/Bacteroidetes ratio (+)	NA	(33)
			Genus level: *Bacteroides* and *Lactobacillus* (+); *Alistipes* and *Lachnospiraceae NK4A136 group* (−)		
Gastroprotection	Ethanol-induced mice	NA, 2 weeks	Phylum level: Bacteroidetes (+); Actinobacteria and Verrucomicrobia (−)	*Dorea*, *Olsenella* and *Bosea*	(41)
			Genus level: *Bacteroides* (+)		
Anti-fatigue	Rotarod test and Treadmill test in mice	2, 4, and 6 mL/kg BW, 4 weeks	Phylum level: Bacteroidetes (+); Firmicutes and Actinobacteria (−)	*Faecalibacterium*, Muribaculaceae, *Coriobacteriaceae_UCG_002*, Desulfobacteria and *Faecalibaculum*	(16)
			Genus level: *Alistipes*, *Alloprevotella*, *Lactobacillus*, *g_UCG-010* and *Muribaculaceae* (+); *Dubosiella* (−)		
Anti-atherosclerosis	High-fat diet-induced ApoE^−/−^ mice	3 and 6 mL/kg BW, 8 weeks	Phylum level: Bacteroidetes and Tenericutes (+); Firmicutes and Firmicutes/Bacteroidetes ratio (−)	*Faecalibaculum*, *Defluviitaleaceae UCG*, *Streptococcus*, *Enterorhabdus, Bilophila* and *Leuconostoc*	(40)
			Genus level: *Alloprevotella* and *Lachnospiraceae NK4A136 groups* (+); *Faecalibaculum*, *Dubosiella*, *Coriobacteriaceae UCG-002* and *Lactobacillus* (−)		
Anti-Alzheimer’s disease	Aluminum chloride-induced rats	1.5 and 3 mL/kg BW, 7 weeks	Lactobacillales (+); Enterobacteriaceae (−)	*Lactobacillus*	(34)
Anti-Alzheimer’s disease	Aβ_25-35_-induced mice	0.5 and 1 mL/kg BW, 4 weeks	Phylum level: Bacteroidetes (+); Firmicutes (−)	NA	(35)
Anti-Alzheimer’s disease	Aluminum chloride-induced rats	2 mL/kg BW, 7 weeks	Family level: Muribaculaceae (+); Lachnospiraceae (−)	NA	(8)
			Genus level: *[Eubacterium]_coprostanoligenes_group*, *Lactobacillus_johnsonii* (+); *Lachnospiraceae_ND3007_group* and *Bacteroides* (−)		
Anti-Alzheimer’s disease	Aluminum chloride-induced rats	3 mL/kg BW, 7 weeks	Phylum level: Bacteroidetes and Proteobacteria (+); Firmicutes (−)	NA	(36)
			Genus level: *Bacteroides pectinophilus group*, *Eubacterium xylanophilum group,* and *Intestinimonas* (+); *Lachnospira* and *Ruminiclostridium* (−)		
Hepatoprotection	Ethanol-induced mice	NA, 4 weeks	Phylum level: Bacteroidota (+); Firmicutes (−)	NA	(42)
			Genus level: *Alloprevotella* and *Bacteroides* (+); *Turicibacter* (−)		
Anti-obese	High-fat diet-induced mice	3 and 6 mL/kg BW, 8 weeks	Phylum level: Bacteroidetes (+); Firmicutes and Firmicutes/Bacteroidetes ratio (−)	Actinobacteria, Lactobacillaceae, Coriobacteriaceae, Bacilli, Coriobacteriia, Coriobacteriales, Lactobacillales, *Anoxybacillus*, *Limnobacter*, *Perlucidibaca*, *Lactobacillus* and *Finegoldia*	(37)
			Genus level: *Lactobacillus*, *Bacteroides* and *Alloprevotella* (+); *Allobaculum*, *Lachnospiraceae NK4A136 group* and *Helicobacter* (−)		
Anti-obese	High-fat diet-induced mice	2 g/kg BW, 8 weeks	Phylum level: Bacteroidetes (+); Firmicutes and Firmicutes/Bacteroidetes ratio (−)	NA	(38)
			Genus level: *Alistipes*, *Blautia*, *Alloprevotella* and *Akkermansia* (+); *Lactobacillus*, *Bacteroides*, *Helicobacter* and *Parabacteroides* (−)		
Hypolipidemic effect	High-fat diet-induced mice	9 g/kg BW, 6 weeks	Phylum level: Bacteroidota and Desulfobacterota (+); Firmicutes and Proteobacteria (−)	NA	(39)
			Genus level: *Dubosiella*, *Lactobacillus* and *Alistipes* (+); *Staphylococcus* and *Aerococcus* (−)		

Regarding anti-Alzheimer’s disease action, camellia oil added Lactobacillales and reduced Enterobacteriaceae, with *Lactobacillus* as the differential gut microbiota biomarker, toward aluminum chloride-induced rats (34). At the phylum level, camellia oil enhanced Bacteroidetes and Proteobacteria and lowered Firmicutes (36). At genus level, camellia oil elevated the abundances of *Bacteroides pectinophilus group*, *Eubacterium xylanophilum group*, *Intestinimonas*, *[Eubacterium]_coprostanoligenes_group* and *Lactobacillus_johnsonii*, and decreased those of *Lachnospira*, *Ruminiclostridium*, *Lachnospiraceae_ND3007_group* and *Bacteroides* (8, 36). On the other hand, camellia oil intervention upregulated Bacteroidetes and downregulated Firmicutes while exerted anti-Alzheimer’s disease action on Aβ_25–35_-induced mice (35).

To anti-obese activity, camellia oil enhanced Bacteroidetes and reduced Firmicutes and Firmicutes/Bacteroidetes ratio in high-fat diet-induced mice, at the phylum level (37, 38). At the genus level, camellia oil increased the abundances of six bacteria (*Lactobacillus*, *Bacteroides*, *Alloprevotella*, *Alistipes*, *Blautia*, etc.), and decreased those of six bacteria (*Allobaculum*, *Lachnospiraceae NK4A136 group*, *Helicobacter*, *Lactobacillus*, *Bacteroides*, etc.) in high-fat diet-induced mice (37, 38). Moreover, 12 types of bacteria (Actinobacteria, Lactobacillaceae, Coriobacteriaceae, Bacilli, Coriobacteriia, etc.) were differential gut microbiota biomarkers for camellia oil that exerted anti-obese effect against high-fat diet-induced mice (37).

Other effects included anti-atherosclerosis (40), gastroprotection (41), hepatoprotection (42), anti-fatigue (16), and hypolipidemic effects (39). Camellia oil upregulated the abundances of Bacteroidetes, Tenericutes, Alloprevotella, and/or Desulfobacterota, and downregulated those of Firmicutes, Actinobacteria, Verrucomicrobia, Turicibacter, and Proteobacteria along with Firmicutes/Bacteroidetes ratio at phylum level, in high-fat diet or ethanol-induced *ApoE^−/−^* mice and/or normal mice. At the genus level, camellia oil enhanced the abundances of nine bacteria (*Bacteroides*, *Alistipes*, *Alloprevotella*, *Lactobacillus*, *g_UCG-010*, etc.) and lowered those of seven bacteria (*Dubosiella*, *Faecalibaculum*, *Coriobacteriaceae UCG-002*, *Lactobacillus*, *Turicibacter*, etc.) (16, 39–42). Moreover, *Dorea*, *Olsenella*, and *Bosea* were identified to be differential gut microbiota biomarkers for camellia oil that exhibited gastroprotection on ethanol-induced mice (41). Six bacteria (*Faecalibaculum*, *Defluviitaleaceae UCG*, *Streptococcus*, *Enterorhabdus*, *Bilophila*, etc.) were differential gut microbiota biomarkers for camellia oil exerted anti-atherosclerosis effect against high-fat diet-induced *ApoE^−/−^* mice (40).

Some differences and similarities in gut microbiota modulation could be found while camellia oil exhibited health-promoting effects in animal experiments. In terms of the similarities, at the phylum level, camellia oil treatment mostly caused increment in Bacteroidetes (16, 35–39, 41, 42) and reduction in Firmicutes (16, 35–39, 42). These led to the decrease of Firmicutes/Bacteroidetes ratio (37–40). Meanwhile, the abundance of Actinobacteria was lowered while camellia oil exerted gastroprotection (41) and anti-fatigue (16) effects. At the genus level, the amount of *Bacteroides* was raised as camellia oil exhibiting anti-ulcerative colitis (33), gastroprotection (41), hepatoprotection (42), and anti-obese (37) effects. The abundance of *Alloprevotella* was elevated, while camellia oil showed anti-fatigue (16), anti-atherosclerosis (40), hepatoprotection (42), and anti-obese (37, 38) activities. That of *Alistipes* was enhanced as camellia oil displayed anti-fatigue (16), anti-obese (38), and hypolipidemic (39) effects. On the contrary, the abundance of *Dubosiella* was decreased as camellia oil revealed anti-fatigue (16) and anti-atherosclerosis (40) activities. That of *Lachnospiraceae NK4A136 group* was reduced, while camellia oil showed anti-ulcerative colitis (33) and anti-obese (37) actions. The amount of *Lactobacillus* was downregulated while camellia oil generated anti-atherosclerosis and anti-obese effects (37, 38).

Regarding the differences, at the phylum level, the abundance of Firmicutes was increased and/or that of Bacteroidetes was decreased, while camellia oil exerted anti-ulcerative colitis action (32, 33). Whereas, opposite phenomena were observed as camellia oil showed gastroprotection (41), anti-fatigue (16), anti-atherosclerosis (40), anti-Alzheimer’s disease (35, 36), hepatoprotection (42), anti-obese (37, 38), and hypolipidemic effects (39). Correspondingly, the Firmicutes/Bacteroidetes ratio was enlarged while camellia oil exerted anti-ulcerative colitis action (33), while it was shrunk as the oil revealed anti-atherosclerosis (40) and anti-obese (37, 38) effects. Meanwhile, the amount of Proteobacteria was added and reduced while camellia oil exhibited anti-Alzheimer’s disease action (36) and hypolipidemic effect (39), respectively. At the genus level, the abundance of *Bacteroides* was decreased while camellia oil showed anti-Alzheimer’s disease (8) and anti-obese (38) effects, whereas it was increased as this oil displayed anti-ulcerative colitis (33), gastroprotection (41), hepatoprotection (42) and anti-obese (37) activities. Meanwhile, the abundance of *Lactobacillus* was upregulated while camellia oil displayed anti-ulcerative colitis (33), anti-fatigue (16), and hypolipidemic (39) effects, and it was downregulated as camellia oil exerted anti-atherosclerosis action (40). That of *Lachnospiraceae NK4A136 group* was decreased while camellia oil exhibited anti-ulcerative colitis (33) and anti-obese (37) activities, and it was increased as the oil showed anti-atherosclerosis action (40). The amount of *Alistipes* was reduced while camellia oil revealed anti-ulcerative colitis effect (33), while it was enhanced as this oil exerted anti-fatigue (16), anti-obese (38), and hypolipidemic (39) effects. The abundance of *Dubosiella* was decreased while camellia oil showed anti-fatigue (16) and anti-atherosclerosis (40) activities, whereas it was increased as the oil exerted hypolipidemic effect (39).

Overall, camellia oil could modulate gut microbiota during their many health-promoting effects. Moreover, differential gut microbiota biomarkers have been screened for it and showed anti-ulcerative colitis, anti-Alzheimer’s disease, anti-obese, and gastroprotection activities. Furthermore, some differences and similarities in gut microbiota modulation have been found while this oil exhibited different health-promoting effects.

### 3.2 Olive oil modulation

**Figure 2:**
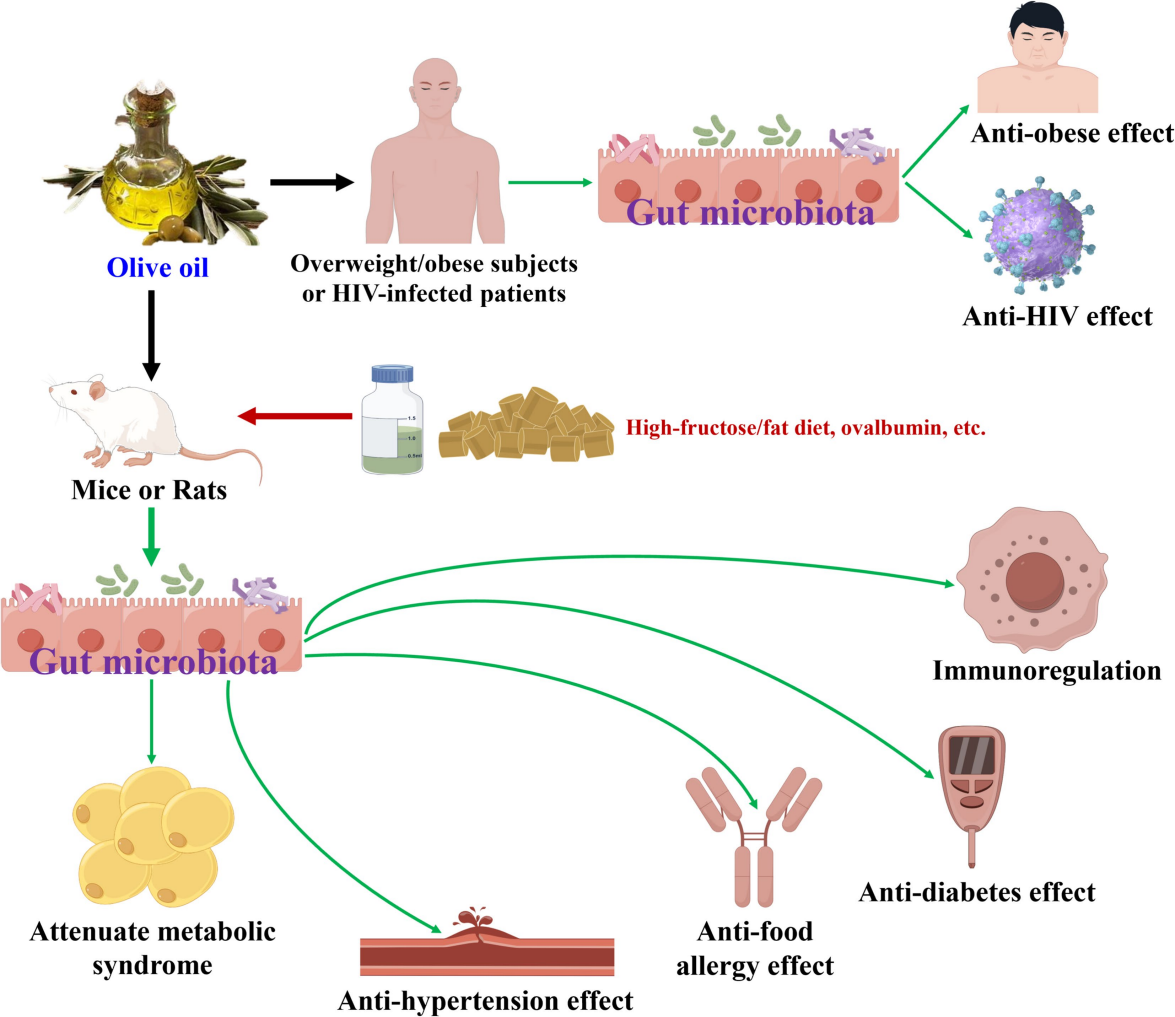
Olive oil modulates gut microbiota during its health-promoting effects.

Olive oil has been proven to modulate gut microbiota during its health-promoting effects (19, 43–50), as illustrated in Table 2 and Figure 2. In terms of attenuate metabolic syndrome action, olive oil increased the abundances of six bacteria (Eubacteriaceae, Bifidobacteriaceae, Anaeroplasmataceae, Erysipelotrichaceae, Clostridiaceae_1, etc.) and decreased those of seven bacteria (Corynebacteriaceae, Enterococcaceae, Aerococcaceae, Staphylococcaceae, Coriobacteriaceae, etc.) at family level in high-fructose/fat diet-induced rats or normal mice (43, 45). At the phylum level, olive oil enhanced the amount of Proteobacteria in normal mice (44). At the genus level, olive oil elevated the amounts of eight bacteria (*Olsenella*, *Bifidobacterium*, *[Eubacterium]_fissicatena_group*, *Ruminococcaceae_UCG-014*, *Allobaculum*, etc.), and lowered those of 12 bacteria (*Bilophila*, *Aerococcus*, *Staphylococcus*, *uncultured_bacterium_f_Coriobacteriaceae*, *Faecalitalea*, etc.) (43–45). Moreover, six types of bacteria (c__Gammaproteobacteria, o__Enterobacteriales, f__Enterobacteriaceae, *s__uncultured_bacterium_g_Blautia*, *s__uncultured_bacterium_g_Allobaculum*, etc.) were screened to be differential gut microbiota biomarkers for olive oil and showed attenuate metabolic syndrome activity against high-fructose/fat diet-induced rats (43).

**Table 2:** Gut microbiota modulation of olive oil during its health-promoting effects.

Health-promoting effect	Experimental model	Oil dosage and treatment time	Gut microbiota modulation	Gut microbiota marker	References
Attenuate metabolic syndrome	High-fructose/fat diet-induced rats	10%, 12 weeks	Family level: Eubacteriaceae, Bifidobacteriaceae, Anaeroplasmataceae, Erysipelotrichaceae, and Clostridiaceae_1 (+); Corynebacteriaceae, Enterococcaceae, Aerococcaceae, Staphylococcaceae and Coriobacteriaceae (−)	c__Gammaproteobacteria, *s__uncultured_bacterium_g_Blautia*, o__Enterobacteriales, f__Enterobacteriaceae, *s__uncultured_bacterium_g_Allobaculum* and *g__Allobaculum*	(43)
			Genus level: *Olsenella*, *Bifidobacterium*, *[Eubacterium]_fissicatena_group*, *Ruminococcaceae_UCG-014* and *Allobaculum* (+); *Bilophila*, *Aerococcus*, *Staphylococcus*, *uncultured_bacterium_f_Coriobacteriaceae*, *Faecalitalea*, *Enterococcus*, *Corynebacterium_1* and *[Ruminococcus]_torques_group* (−)		
Attenuate metabolic syndrome	Normal mice	20%, 12 weeks	Phylum level: Proteobacteria (+)	NA	(44)
			Genus level: *Parasutterella*, *Marispirillum* and *Marinilabilia* (+); *Prevotella*, *Anaerophaga*, *Fusicatenibacter* and *Christensenella* (−)		
Attenuate metabolic syndrome	Normal mice	20%, 12 weeks	Family level: Erysipelotrichaceae and Sutterellaceae (+); Prevotellaceae and Christensenellaceae (−)	NA	(45)
			Genus level: *Parasutterella* and *Marinilabilia* (+); *Anaerophaga* and *Fusicatenibacter* (−)		
Anti-hypertension	Spontaneously hypertensive rats	20%, 12 weeks	*Lactobacillus* sp., *Clostridia XIVa* and *Universal* (+)	NA	(46)
Anti-food allergy	Ovalbumin-sensitized mice	1.0, 2.0 and 3.0 g/kg BW, 7 weeks	Phylum level: Actinobacteriota (+)	f__Bifidobacteriaceae, o__Bifidobacteriales, o__Micrococcales, c__Actinobacteria, f__Streptococcaceae, f__Staphylococcaceae, o__Staphylococcales, f__ Enterobacteriaceae, o__Enterobacterales, f__Pseudomonadaceae and o__Pseudomonadales	(47)
			Genus level: *Clostridiaceae* (+); *Burkholderiaceae* (−)		
Anti-diabetes	NOD/LtJ mice	2.5 mL/kg BW, 14 weeks	Phylum level: Bacteroidetes, Verrucomicrobia, Cyanobacteria, and Bacteroidetes/Firmicutes ratio (+); Firmicutes (−)	*Bacteroides*, Muribaculaceae, *Alistipes*, *Lachnoclostridium*, *Tyzzerella*, *Ruminococcaceae_UCG_005*, *Eubacterium_xylanophilum_group*, *Intestinimonas*, *Angelakisella*, *Akkermansia* and *Gastranaerophilales*	(48)
			Genus level: *Bacteroides*, *Muribaculum*, *Akkermansia*, *Ruminococcaceae_UCG_005*, *Intestinimonas* and *Angelakisella* (+); *Lachnospira* and *Eubacterium_xylanophilum_group* (−)		
Anti-obese	Overweight/obese subjects	40 g/die, 3 months	Lactic acid bacteria (+)	NA	(19)
Anti-HIV	HIV-infected patients	50 mL, 12 weeks	Genus level: *Gardnerella* (+); *Mogibacterium*, *Dethiosulfovibrionaceae* and *Coprococcus* (−)	*g__Gardnerella*	(49)
Immunoregulation	Normal mice	157.8 g, 10 weeks	Phylum level: Deferibacteres (+); Firmicutes (−)	NA	(50)
			Genus level: *Mucispirillum*, *Lachnospiraceae*, *Bacteroides*, *Allobaculum* and *Coriobacteriaceae* spp. (+); *S24-7* spp. (−)		

Regarding the other above-mentioned health-promoting effects, olive oil boosted the abundances of Actinobacteria, Bacteroidetes, Verrucomicrobia, Cyanobacteria, and Deferibacteres as well as Bacteroidetes/Firmicutes ratio, and declined that of Firmicutes at the phylum level (47, 48, 50). At the genus level, olive oil raised the amounts of 12 bacteria (*Clostridiaceae*, *Bacteroides*, *Muribaculum*, *Akkermansia*, *Ruminococcaceae_UCG_005*, etc.), and reduced those of seven bacteria (*Burkholderiaceae*, *Lachnospira*, *Eubacterium_xylanophilum_group*, *Mogibacterium*, *Dethiosulfovibrionaceae*, etc.), while exerted anti-food allergy, anti-diabetes, anti-HIV, and immunoregulation effects (47–50). On the other hand, olive oil supplement added the abundances of *Lactobacillus* sp., *Clostridia XIVa*, and *Universal* as exerted anti-hypertension activity (46), and aggrandized the number of Lactic acid bacteria while exhibited anti-obese action (19). Moreover, for olive oil revealed anti-food allergy effect, 11 types of bacteria (f__Bifidobacteriaceae, o__Bifidobacteriales, o__Micrococcales, c__Actinobacteria, f__Streptococcaceae, etc.) were identified to be differential gut microbiota biomarkers (47). Olive oil exhibited anti-diabetes activity, and 11 types of bacteria (*Bacteroides*, Muribaculaceae, *Alistipes*, *Lachnoclostridium*, *Tyzzerella*, etc.) were characterized as differential gut microbiota biomarkers (48). As to olive oil showed anti-HIV action, *g__Gardnerella* was the differential gut microbiota biomarker (49).

Some similarities in gut microbiota modulation could be seen while olive oil exhibited different health-promoting effects in animal experiments. At the phylum level, the abundance of Firmicutes was reduced and that of Bacteroides was enhanced, while olive oil exerted anti-diabetes and immunoregulation effects (48, 50).

In short, olive oil could modulate gut microbiota during their health-promoting effects. Moreover, differential gut microbiota biomarkers have been gained for exerting attenuate metabolic syndrome, anti-food allergy, anti-diabetes, and anti-HIV actions. Furthermore, some similarities in gut microbiota modulation have been discovered as this oil exhibited anti-diabetes and immunoregulation effects.

### 3.3 Flaxseed oil modulation

**Figure 3:**
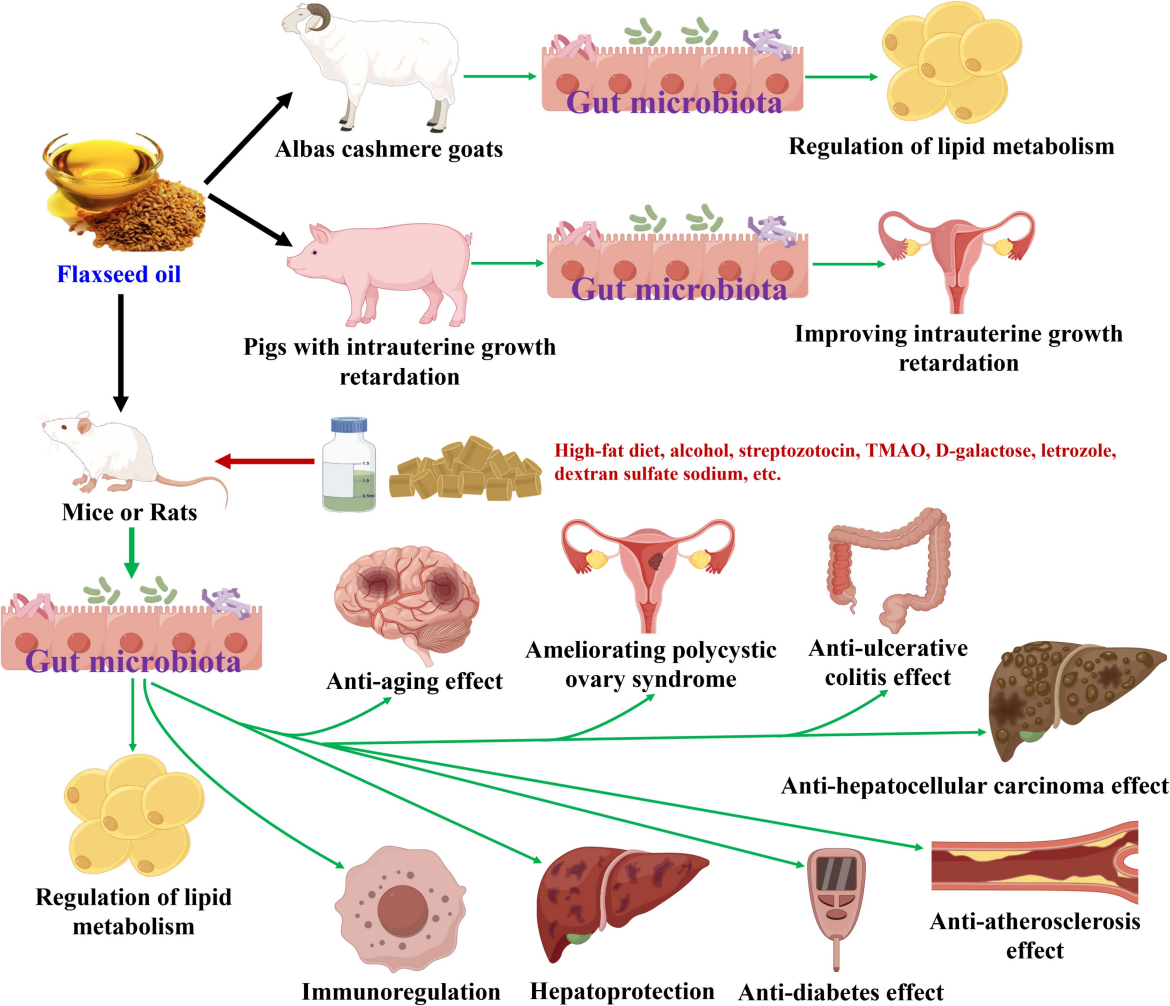
Flaxseed oil modulates gut microbiota during its health-promoting effects.

Flaxseed oil has been discovered to modulate gut microbiota during its health-promoting effects (18, 50–63), as displayed in Table 3 and Figure 3. In terms of regulation of lipid metabolism effect, flaxseed oil treatment increased the abundances of Actinobacteria, Proteobacteria, and Spirochaeta, and decreased those of Firmicutes, Saccharibacteria, and Verrucomicrobia at the phylum level in Albas cashmere goats (52, 53). At the genus level, flaxseed oil elevated the amounts of 17 bacteria (*Uc_Ruminococcaceae*, *Uc_Lachnospiraceae*, *Oscillospira*, *Ruminococcus*, *Ruminococcus_2*, etc.), and declined those of 10 bacteria (*Sporosarcina*, *Mogibacteriaceae*, *Clostridium*, *unclassified_f_Peptostreptococcaceae*, *Clostridium_sensu_stricto_1*, etc.) (51–53). Moreover, six types of bacteria (*Ruminococcus*, *Anaerotruncus*, Ruminococcaceae, Lachnospiraceae, *Dehalobacterium*, etc.) have been screened to be differential gut microbiota biomarkers for flaxseed oil and showed lipid metabolism regulation against high-fat diet-induced mice (51). While *Lachnospiraceae_NK3A20_group* has been identified as a differential gut microbiota biomarker for flaxseed oil-regulated lipid metabolism in Albas cashmere goats (53). Regarding anti-diabetes action, flaxseed oil supplement enhanced Bacteroidetes, and reduced Firmicutes and Firmicutes*/*Bacteroidetes ratio at phylum level in streptozotocin-nicotinamide-induced rats (55). At the genus level, flaxseed oil upregulated *Alistipes* and downregulated *Blautia*. While the study of Xia et al. (56) has revealed that flaxseed oil treatment raised the abundance of six bacteria (*Bacteroidetes*, *Muribaculaceae*, *Streptococcaceae*, *Lactococcus*, *Streptococcus*, etc.). To anti-atherosclerosis activity, flaxseed oil lessened the number of Eubacterium and Firmicutes*/*Bacteroidetes ratio at the phylum level in TMAO and high-fat diet-induced *ApoE*^−/−^ mice (57, 58). At the genus level, flaxseed oil expanded the abundances of *Alistipes* and *Odoribacter* and diminished the amounts of seven bacteria (*Intestinimonas*, *Bilophila*, *Anaerotruncus*, *Oscillibacter*, *Lachnoclostridium*, etc.).

**Table 3:** Gut microbiota modulation of flaxseed oil during its health-promoting effects.

Health-promoting effect	Experimental model	Oil dosage and treatment time	Gut microbiota modulation	Gut microbiota marker	References
Immunoregulation	Normal mice	157.8 g, 10 weeks	Phylum level: Firmicutes (−)	NA	(50)
			Genus level: *Allobaculum* and *Coriobacteriaceae* spp. (+); *Clostridi ales* spp. (−)		
Regulation of lipid metabolism	High-fat diet-induced mice	30, 60 and 90%, 8 weeks	Genus level: *unclassified_Ruminococcaceae*, *unclassified_Lachnospiraceae*, *Oscillospira* and *Ruminococcus* (+); *unclassified_S24-7*, *Sporosarcina*, *Mogibacteriaceae* and *Clostridium* (−)	*Ruminococcus*, *Anaerotruncus*, Ruminococcaceae, Lachnospiraceae, *Dehalobacterium*, and Eubacteriaceae	(51)
Regulation of lipid metabolism	Albas cashmere goats	36%, 90 d	Phylum level: Actinobacteria (+)	NA	(52)
			Genus level: *Ruminococcus_2*, *Ruminococcaceae_UCG-014*, *Christensenellaceae_R-7_group*, *[Eubacterium]_coprostanoligenes_group*, *Turicibacter*, *Family_XIII_AD3011_group*, *Lactobacillus*, *Aeriscardovia*, *Bifidobacterium*, *Olsenella* and *Ureaplasma* (+); *unclassified_f_Peptostreptococcaceae*, *Clostridium_sensu_stricto_1*, *Lachnospiraceae_NK3A20_group*, *Acetitomaculum*, *Streptococcus*, *[Ruminococcus]_gauvreauii_group* and *Mycoplasma* (−)		
Regulation of lipid metabolism	Albas cashmere goats	2.0% or 2.5%, 90 d	Phylum level: Proteobacteria and Spirochaeta (+); Firmicutes, Saccharibacteria and Verrucomicrobia (−)	*Lachnospiraceae_NK3A20_group*	(53)
			Genus level: *Rikenellaceae_RC9_gut_group* and *Lachnospiraceae_NK3A20_group* (+)		
Hepatoprotection	Alcohol-induced liver injury in mice	39.6 g/L, 6 weeks	Phylum level: Proteobacteria (−)	NA	(59)
			Genus level: *Parabacteroides* (+)		
Anti-diabetes	Streptozotocin-nicotinamide-induced rats	10%, 5 weeks	Phylum level: Bacteroidetes (+); Firmicutes and Firmicutes*/*Bacteroidetes ratio (−)	NA	(55)
			Genus level: *Alistipes* (+); *Blautia* (−)		
Anti-diabetes	Streptozotocin-induced mice	7.0 and 10.5%, 6 weeks	*Bacteroidetes*, *Muribaculaceae*, *Streptococcaceae*, *Lactococcus*, *Streptococcus* and *uncultured_bacterium_f_Muribaculaceae* (+)	NA	(56)
Anti-atherosclerosis	TMAO-induced *ApoE*^−/−^ mice	102.1 g, 12 weeks	Phylum level: Eubacterium (−)	NA	(57)
			Genus level: *Alistipes* and *Odoribacter* (+)		
Anti-atherosclerosis	High-fat diet-induced *ApoE*^−/−^ mice	10%, 12 weeks	Phylum level: Firmicutes*/*Bacteroidetes ratio (−)	NA	(58)
			Genus level: *Intestinimonas*, *Bilophila*, *Anaerotruncus*, *Oscillibacter*, *Lachnoclostridium*, *Enterorhabdus* and *Negativibacillus* (−)		
Anti-aging	D-galactose-induced rats	40 g/kg, 8 weeks	Genus level: *uncultured_bacterium_f_Lachnospiraceae* and *Ruminococcaceae_UCG-005* (+); *uncultured_bacterium_f_Desulfovibrionaceae*, *Akkermansia*, *Romboutsia*, *Prevotella_9* and *[Eubacterium]_oxidoreducens_group* (−)	p_Firmicutes, o_Clostridiales, c_Clostridia, f_Lachnospiraceae, *s_uncultured_bacterium_f_Lachnospiracea*, *g_uncultured_bacterium_f_Lachnospiraceae*, *s_uncultured_bacterium_g_Lachnospiraceae_NK4A136_group*, *s_uncultured_bacterium_g_Lactobacillus*, f_Lactobacillaceae, *g_Lactobacillus* and o_Lactobacillales	(60)
Ameliorating polycystic ovary syndrome	Letrozole-induced rats	1 mL/kg, 8 weeks	Phylum level: Actinobacteria, Proteobacteria, and Firmicutes/Bacteroidetes ratio (−)	NA	(61)
			Genus level: *Lactobacillus*, *Allobaculum*, *Butyrivibrio*, *Desulfovibrio*, *Bifidobacterium*, *Faecalibacterium* and *Parabacteroides* (+); *Bacteroides* and *Streptococcus* (−)		
Improving intrauterine growth retardation	Pigs with intrauterine growth retardation	4%, 3 weeks	Phylum level: Actinobacteria and Melainabacteria (+); Spirochaetes (−)	NA	(62)
			Genus level: *Bifidobacterium* and *Blautia* (+)		
Anti-ulcerative colitis	Dextran sulfate sodium-induced colitis in rats	400, 800 and 1,600 mg/kg BW, 6 weeks	Phylum level: Bacteroidetes, Proteobacteria, and Verrucomicrobia (+)	NA	(63)
			Genus level: *Lactobacillus*, *Lachnospiraceae_NK4A136_group*, *Lachnoclostridium*, *Phascolarctobacterium* and *Prevotellaceae_UCG-001* (+); *Romboutsia* and *Ruminococcaceae_UCG-005* (−)		
Anti-hepatocellular carcinoma	Orthotopic hepatocellular carcinoma mice	NA, 30 d	Phylum level: Firmicutes (+); Proteobacteria and Actinobacteriota (−)	NA	(18)
			Genus level: *norank_f_norank_o_Clostridia UCG-014*, *norank_f_norank_Clostridia_vadinBB60_group* and *Turicibacter* (+); *Escherichia-shigella*, *Enterorhabdus* and *Lactococcus* (−)		
Improving lipid metabolism and gut barrier homeostasis	Normal rats	5 g/kg BW, 8 weeks	Family level: Lactobacillaceae and Ruminococcaceae (−)	NA	(54)
			Species level: *uncultured_bacterium lahnospiraceae* (+); *Lactobacillus* (−)		

For aforementioned other effects, flaxseed oil aggrandized the abundances of Actinobacteria, Melainabacteria, Bacteroidetes, Proteobacteria, Verrucomicrobia, and/or Firmicutes and lowered those of Firmicutes, Proteobacteria, Actinobacteria and/or Spirochaetes as well as Firmicutes*/*Bacteroidetes ratio, at the phylum level (18, 50, 59, 61–63). At genus level, flaxseed oil increased the amounts of 19 bacteria (*Allobaculum*, *Coriobacteriaceae* spp., *Parabacteroides*, *uncultured_bacterium_f_Lachnospiraceae*, *Ruminococcaceae_UCG-005*, etc.) and decreased those of 12 bacteria (*Clostridiales* spp., *uncultured_bacterium_f_Desulfovibrionaceae*, *Akkermansia*, *Romboutsia*, *Prevotella_9*, etc.). Moreover, for the anti-aging action of flaxseed oil on D-galactose-induced rats, 11 types of bacteria (p_Firmicutes, o_Clostridiales, c_Clostridia, f_Lachnospiraceae, *s_uncultured_bacterium_f_Lachnospiracea*, etc.) have been identified to be differential gut microbiota biomarkers (60).

Some similarities and differences in gut microbiota modulation could be observed, while flaxseed oil showed different health-promoting effects in animal experiments. At the phylum level, the abundance of Firmicutes was downregulated while flaxseed oil exerted immunoregulation (50), regulation of lipid metabolism (53), and anti-diabetes (55) effects. Meanwhile, Firmicutes*/*Bacteroidetes ratio was diminished as this oil exhibited anti-diabetes (55), anti-atherosclerosis (58), and ameliorating polycystic ovary syndrome (61) activities. While that of Firmicutes was upregulated as flaxseed oil revealed anti-hepatocellular carcinoma action (18). The amount of Bacteroidetes was added while flaxseed oil displayed anti-diabetes (55) and anti-ulcerative colitis (63) effects. The number of Actinobacteria was increased while flaxseed oil reflected regulation of lipid metabolism (52) and improving intrauterine growth retardation (62) effects, whereas it was decreased as this oil showed ameliorating polycystic ovary syndrome action (61). The abundance of Proteobacteria was raised while flaxseed oil exerted regulation of lipid metabolism (53) and anti-ulcerative colitis (63) effects, while it was shrunk as flaxseed oil exhibited hepatoprotection (59), ameliorating polycystic ovary syndrome (61) and anti-hepatocellular carcinoma (18) actions. The amount of Verrucomicrobia was reduced while flaxseed oil revealed regulation of lipid metabolism effect (53), while it was elevated as this oil emerged anti-ulcerative colitis action (63). At the genus level, the number of *Allobaculum* was augmented, while flaxseed oil showed immunoregulation (50) and ameliorating polycystic ovary syndrome (61) effects. The amount of *Lachnoclostridium* was downregulated while flaxseed oil revealed anti-atherosclerosis effect (58), and that was upregulated as the oil showed anti-ulcerative colitis action (63). The number of *Alistipes* was enhanced as flaxseed oil displayed anti-diabetes (55) and anti-atherosclerosis (57) activities. The abundance of *Enterorhabdus* was declined while flaxseed oil exerted anti-atherosclerosis (58) and anti-hepatocellular carcinoma (18) actions. The amount of *Romboutsia* was decreased as flaxseed oil exhibited anti-aging (60) and anti-ulcerative colitis (63) activities. The abundance of *Ruminococcaceae_UCG-005* was upregulated while flaxseed oil showed anti-aging effect (60) and was downregulated as this oil exhibited anti-ulcerative colitis activity (63). The quantity of *Lactobacillus* was enhanced while flaxseed oil exerted ameliorating polycystic ovary syndrome (61) and anti-ulcerative colitis (63) effects. The number of *Bifidobacterium* was enhanced, while flaxseed oil revealed regulation of lipid metabolism (52), ameliorating polycystic ovary syndrome (61), and improving intrauterine growth retardation (62) activities. The abundance of *Blautia* was decreased while flaxseed oil exhibited anti-diabetes effect (55), while it was increased as the oil exerted improving intrauterine growth retardation action (62). The amount of *Streptococcus* was declined, while flaxseed oil showed regulation of lipid metabolism (52) and ameliorating polycystic ovary syndrome (61) activities. The number of *Turicibacter* was added while flaxseed oil displayed regulation of lipid metabolism (52) and anti-hepatocellular carcinoma (18) effects. The abundance of *Enterorhabdus* was lowered while flaxseed oil reflected anti-atherosclerosis (58) and anti-hepatocellular carcinoma (18) actions.

In a word, flaxseed oil could modulate gut microbiota during their many health-promoting effects. Moreover, differential gut microbiota biomarkers have been acquired for their revealed lipid metabolism regulation and anti-aging effect. Furthermore, some differences and similarities in gut microbiota modulation have been found while this oil exhibited different health-promoting effects.

### 3.4 Other oil modulations

**Figure 4:**
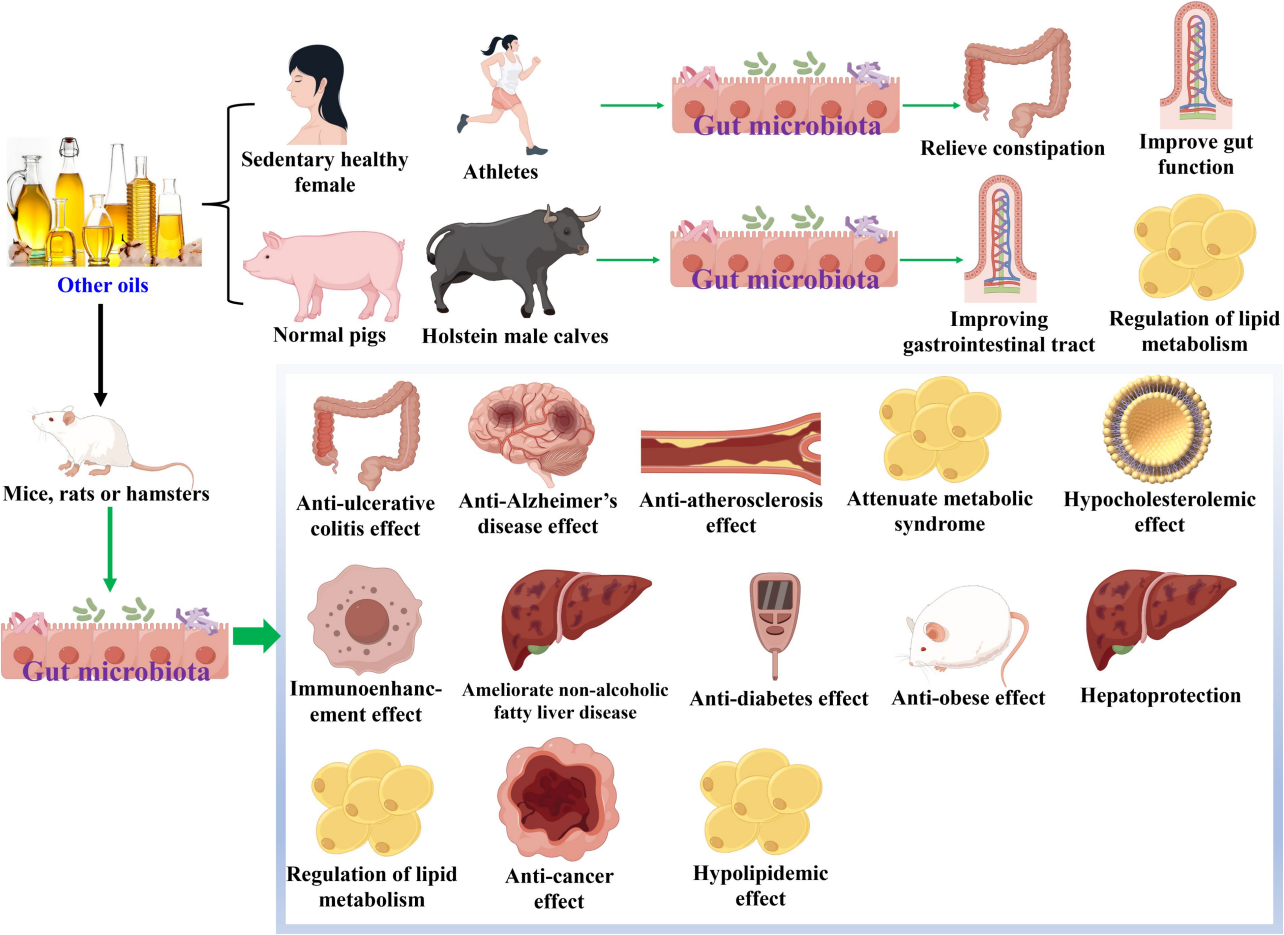
Other oils modulate gut microbiota during their health-promoting effects.

There were other edible plant oils which could modulate gut microbiota during their health-promoting effects, as shown in Table 4 and Figure 4. In terms of walnut oil, it increased the abundances of S24-7, Lachnospiraceae and Ruminococcaceae, and decreased that of Moraxellaceae at family level while exerted anti-Alzheimer’s disease effect on scopolamine-induced mice (64). At the phylum level, walnut oil elevated the amounts of Firmicutes, Actinobacteria, and/or Bacteroidetes, and declined those of Proteobacteria and/or Epsilonbacteraeota while revealed anti-ulcerative colitis action in dextran sulfate sodium-induced colitis mice (65) and anti-Alzheimer’s disease activity in scopolamine-induced mice (64). At the genus level, walnut oil enhanced the quantities of seven bacteria (*Lactobacillus*, *Lachnospiraceae_NK4A136_group*, *Faecalibaculum*, *Bifidobacterium*, *[Ruminococcus]_torques_group*, etc.), and reduced those of six bacteria (*Helicobacter*, *Bacteroides*, *Clostridium_sensu_stricto_1*, *Staphylococcus*, *Desulfovibrio*, etc.) (65). Moreover, Ruminococcaceae and Mogibacteriaceae were chosen to be differential gut microbiota biomarkers for walnut oil, which exhibited anti-Alzheimer’s disease effect against scopolamine-induced mice (64).

**Table 4:** Gut microbiota modulation of other oils during their health-promoting effects.

Oil	Health-promoting effect	Experimental model	Oil dosage and treatment time	Gut microbiota modulation	Gut microbiota marker	References
Walnut oil	Antioxidant, anti-inflammatory and immunoregulation	Normal mice	2.5, 5 and 10 mL/kg, 4 weeks	Genus level: *Lactobacillus* (+); *Helicobacter* (−)	NA	(101)
Walnut oil	Anti-ulcerative colitis	Dextran sulfate sodium-induced colitis mice	2.5 mL/kg, 4 weeks	Phylum level: Firmicutes and Actinobacteria (+); Proteobacteria and Epsilonbacteraeota (−)	NA	(65)
				Genus level: *Lactobacillus*, *Lachnospiraceae_NK4A136_group*, *Faecalibaculum*, *Bifidobacterium*, *[Ruminococcus]_torques_group*, *Akkermansia* and *Roseburia* (+); *Bacteroides*, *Helicobacter*, *Clostridium_sensu_stricto_1*, *Staphylococcus*, *Desulfovibrio* and *Streptococcus* (−)		
Walnut oil	Anti-Alzheimer’s disease	Scopolamine-induced mice	1.63, 3.25 and 16.25 g/kg, 8 weeks	Phylum level: Firmicutes and Bacteroidetes (+); Proteobacteria (−)	Ruminococcaceae and Mogibacteriaceae	(64)
				Family level: S24-7, Lachnospiraceae and Ruminococcaceae (+); Moraxellaceae (−)		
Soybean oil	Anti-atherosclerosis	Normal mice	80 and 160 mg, 4 weeks	Phylum level: Bacteroidetes, Deferribacteres and Proteobacteria (+); Firmicutes, Tenericutes and TM7 (−)	*Prevotella*, *unclassified S24-7*, *Mucispirillum*, *Dehalobacterium*, *Ruminococcus*, *unclassified Peptococcaceae*, *Coprobacillus*, *Bilophila*, *Desulfovibrio*, *Anaeroplasma* and Proteobacteria	(66)
Soybean oil	Attenuate metabolic syndrome	High-fat diet-induced mice	25%, 2 or 8 weeks	*Akkermansia muciniphila*, *Turicibacter*, *Thermicanus*, *Clostridium saccharogumia*, and *Lactobacillus pontis* (+); *Coprobacillus* (−)	NA	(67)
Soybean oil	Increase insulin sensitivity and prevent fatty liver	Normal mice	7% or 21%, 3 months	*Akkermansia muciniphila* and *Faecalibacterium prausnitzii* (+)	NA	(68)
Sea buckthorn seed oil	Hypocholesterolemic effect	High-cholesterol diet-induced hamsters	50 and 100%, 6 weeks	Phylum level: Bacteroidetes (+); Firmicutes and Firmicutes/Bacteroidetes ratio (−)	NA	(69)
				Genus level: *norank_f_Bacteroidales_S24-7_group*, *Allobaculum* and *norank_o_Mollicutes_RF9* (+); *Acietatifactor*, *Lactobacillus*, and *norank_f_Ruminococcaceae* (−)		
Sea buckthorn pulp oil	Immunoenhancement effect	Cyclophosphamide-induced mice	5 and 10 mL/kg, 3 weeks	Genus level: *Alistipes*, *Bacteroides*, *Anaerotruncus*, *Lactobacillus*, *ASF356*, *Roseburia* (+); *Mucispirillum*, *Anaeroplasma*, *Pelagibacterium*, *Brevundimonas*, *Ochrobactrum*, *Acinetobacter*, *Ruminiclostridium*, *Blautia*, *Ruminiclostridium*, *Oscillibacter* and *Faecalibaculum* (−)	Tannerellaceae and *Parabacteroides*	(70)
Sea buckthorn seed and pulp oil	Ameliorate non-alcoholic fatty liver disease	High-fat diet-induced mice	23.6%, 12 weeks	*Oscillibacter* and *Roseburia* (+); *Lactobacillus* (−)	NA	(71)
Coconut oil	Improve diabetes	Alloxan-induced rats	20%, 16 weeks	Phylum level: Firmicutes, Actinobacteria, and Firmicutes*/*Bacteroidetes ratio (+); Bacteroidetes and Spirochaetes (−)	NA	(72)
				Genus level: *Lactobacillus* and *Allobaculum* (+); *Treponema* and *Turicibacter* (−)		
Coconut oil	Improving gastrointestinal tract	Normal pigs	3 g/kg, 2 weeks	*Alloprevotella*, Bifidobacteriales and *Lactobacilli* (+); *Corynebacterium*, Pseudomonadales, *Psychrobacter*, and *Mitsuokella* (−)	NA	(73)
Coconut oil	Anti-obese	Obese rats	0.01 and 0.02%, 4 weeks	Phylum level: Bacteroidetes (+); Firmicutes (−)	NA	(74)
Coconut oil	Anti-obese	Obese rats	NA, 8 weeks	Enterobacteriaceae, *Escherichia* and *Enterococcus* (+)	NA	(75)
Coconut oil	Anti-obese	High-sugar diet-induced rats	1,000 mg/kg, 3 weeks	*E. coli* (−)	NA	(17)
Coconut oil	Regulation of lipid metabolism	Holstein male calves	NA, 6 weeks	Phylum level: Actinobacteria (+)	NA	(76)
				Genus level: *Erysipelotrichaceae_UCG-006* (+)		
Perilla oil	Anti-diabetes	High-fat diet-induced KKAy mice	0.67, 1.33 and 2.00 g/kg BW, 6 weeks	Genus level: *Alistipes*, *Alloprevotella*, *Parabacteroides* and *Rikenella* (+); *unidentified_Ruminococcaceae*, *Lachnoclostridium unidentified_Lachnospiraceae*, *Oscillibacter*, *Blautia*, *Desulfovibrio*, *Angelakisella* and *Bilophila* (−)	NA	(79)
Perilla oil	Anti-non-alcoholic fatty liver disease	High-fat diet-induced rats	5.5%, 16 weeks	Phylum level: Bacteroidetes, Spirochaetes and Verrucomicrobia (+); Firmicutes and Firmicutes*/*Bacteroidetes ratio (−)	NA	(78)
				Genus level: *Roseburia*, *Prevotella*, *Bacteroides* and *Akkermansia* (+); *Ruminococcus*, *Oscillospira* and *Clostridium* (−)		
Perilla oil	Relieve constipation	Sedentary healthy female	9 g, 10 months	Family level: Clostridiaceae (+)	NA	(21)
Perilla oil	Improve gut function	Athletes	3 g and 9 g, 8 weeks	Phylum level: Bacteroidetes (+); Firmicutes, Proteobacteria and Firmicutes/Bacteroidetes ratio (−)	NA	(77)
				Family level: Lachnospiraceae (+)		
Peony seed oil	Hypocholesterolemic effect	High-cholesterol diet-induced hamsters	50 and 100%, 6 weeks	Phylum level: Firmicutes/Bacteroidetes ratio (−)	NA	(80)
				Genus level: *unclassified_f_Ruminococcaceae* and *Ruminococcus_2* (+); *unclassified_f__Erysipelotrichaceae*, *Christensenellaceae_R-7_group*, *norank_o__Mollicutes_RF9*, *Peptococcus*, *norank_f__Eubacteriaceae* and *unclassified_f__Coriobacteriaceae*		
Peony seed oil	Alleviate hyperlipidemia and hyperglycemia	High-fat diet-induced mice	0.5, 1.0 and 1.5 mL, 4 weeks	Phylum level: Proteobacteria and Deferribacteres (−)	*Bacteroides*, *Turicibacter* and *Allobaculum*	(81)
				Genus level: *Lactobacillus*, *Prevotella* and *Parabacteroides* (+); *Ruminococcaceae_Ruminococcu*, *Mucispirillum*, *Oscillospira* and *Coprococcus* (−)		
*Decaisnea insignis* seed oil	Hepatoprotection	Alcohol-induced mice	3, 6 and 12 g/kg, 12 weeks	Phylum level: Saccharibacteria (+)	*Lactobacillus_gasseri*	(82)
				Genus level: *Lactobacillus*, *Conexibacter*, *Pepeococcus*, and *Ruminoccoceae_UCG_004* (+)		
*Decaisnea insignis* seed oil	Hepatoprotection	L-carnitine-induced mice	6 g/kg, 12 weeks	Phylum level: Bacteroidetes (+); Firmicutes and Proteobacteria (−)	NA	(83)
				Genus level: *Akkermansia*, *Lactobacillaceae*, *Bacteroides*, and *Rikenellaceae* (+); *Helicobacter* and *Erysipelotrichaceae* (−)		
Sacha inchi oil	Hypolipidemic effect	High-fat diet-induced rats	0.5, 1.0 and 1.5 mL/kg, 8 weeks	Phylum level: Firmicutes (+); Bacteroidetes (−)	*Roseburia*, *Turicibacter*, *Butyrivibrio*, *Unidentified Enterobacteriaceae*, *Escherichia*, and *Bacteroides*	(84)
				Genus level: *Alistipes* (+); *Unidentified Enterobacteriaceae*, *Bacteroides* and *Lachnoclostridium* (−)		
Sacha inchi oil	Regulation of lipid metabolism	High-fat diet-induced mice	100 and 200 mg/kg, 45 d	Phylum level: Bacteroidetes (+); Firmicutes and Firmicutes/Bacteroidetes ratio (−)	Deferribacteraceae, Deferribacterales, and Deferribacteres	(85)
*Schizochytrium* sp. L. oil					Rhodospirillales and Alphaproteobacteria	
Millet bran oil	Attenuate metabolic syndrome	High-fat diet-induced mice	2 g/kg, 12 weeks	Phylum level: Verrucomicrobia and Saccharibacteria (+)	*Akkernansia*, *PrevotellaceaeUCG_001*, *Erysipelatoclostridium*, *Ruminococcaceae UCG_009*, *unclassified_f__Lachnospiraceae, Butyricimonas*, *Sulfuricurvum*, and *Arcobacter*	(86)
Riceberry bran oil	Anti-cancer	Diethylnitrosamine and 1,2-dimethylhydrazine-induced rats	100 mg/kg, 10 weeks	Phylum level: Bacteroidetes (+); Firmicutes and Firmicutes/Bacteroidetes ratio (−)	NA	(87)
				Genus level: *Ruminococcaceae UCG-013*, *Ruminococcaceae UCG-014*, *Adlercreutzia*, *Enterorhabdus*, *Papillibacter* and *Lachnospiraceae NK4A136 groups* (+); *Eubacterium coprostanoligenes*, *Ruminoclostridium 6* and *Bacteroides* (−)		
*Torreya grandis* oil	Anti-obese	High-fat diet-induced mice	250, 550 and 850 mg/kg, 8 weeks	Phylum level: Firmicutes and Actinobacteria (+); Bacteroidetes and Proteobacteria (−)	NA	(88)
				Family level: Erysipelotrichaceae, Coriobacteriaceae, Lactobacillaceae, and Bifidobacteriaceae (+); Porphyromonadaceae (−)		
*Torreya grandis* oil	Anti-Alzheimer’s disease	Scopolamine-induced mice	1,000 and 3,000 mg/kg, 30 d	Phylum level: Firmicutes/Bacteroidetes ratio (+); Verrucomicrobiota (−)	NA	(89)
				Species level: *Allobaculum*, *Bifidobacterium*, *Olsenella*, *Parasutterella*, *unclassified Ruminococcaceae*, *the siraeum group of Eubacterium*, *Anaerotignum* and *Anaerovorax* (+)		
Safflower oil	Improve glucose intolerance	High fat/high sucrose diet-induced mice	NA, 40 weeks	Phylum level: Deferribacteres (+); Bacteroidetes, Proteobacteria and Actinobacteria (−)	NA	(90)
				Genus level: *Blautia* (+); *Barnesiella* (−)		
Peanut oil	Attenuate metabolic syndrome	High-fat/high sucrose diet-induced rats	10%, 12 weeks	Phylum level: Firmicutes/Bacteroidetes ratio (+)	*g__Faecalibaculum*, *s__uncultured_bacterium_g_Faecalibaculum*, p__Proteobacteria, *g__Ruminococcaceae_UCG_014*, *s__uncult ured_bacterium_g_Ruminococcaceae_UCG_014*, *g__Klebsiella*, *s__uncultured_bacterium_g_Clostridium_sensu_stricto_1*, f__Clostridiaceae_1, *g__Clostridium_sensu_stricto_1* and f__Peptostreptococcaceae	(43)
				Genus level: *Olsenella, Peptoclostridium, Ruminococcaceae_UCG-009, Weissella, Bifidobacterium, [Eubacterium]_fissicatena_group, [Eubacterium]_coprostanoligenes_group, Ruminococcaceae_NK4A214_group, Clostridium_sensu_stricto_1, Ruminococcaceae_UCG-014* and *Faecalibaculum* (+); *Bilophila, Leuconostoc, [Eubacterium]_nodatum_group, Lactococcus, uncultured_bacterium_f_Coriobacteriaceae, Streptococcus, Rothia, [Ruminococcus]_torques_group, Bacteroides, Lachnoclostridium* and *Blautia* (−)		
Kiwifruit seed oil	Anti-obese	High-fat diet-induced mice	1.0 and 3.0 mL/kg, 12 weeks	Phylum level: Bacteroidetes (+); Firmicutes and Firmicutes/Bacteroidetes ratio (−)	*Pseudoflavonifractor*, *Flavonifractor*, *Intestinimonas*, *Romboutsia* and *Olsenlla*	(91)
				Genus level: *Bacteroides*, *Barnesiella*, *Intestinimonas*, *Tannerella*, *Coprobacter*, *Alistipes*, *Odoribacter*, *Alloprevotella*, *Parabacteroides*, *Ruminococcus_2*, *Acetobacteroides*, *Macellibacteroides*, *Stomatobaculum* and *Clostridium XVII* (+); *Bilophila*, *Clostridium IV*, *Acetatifactor*, *Helicobacter*, *Clostridium XIVa*, *Lachnospiracea incertae sedis*, *Akkermansia*, *Mucispirillum*, *Anaerotruncus*, *Eisenbergiella*, *Hydrogenoanaerobacteruim*, *Lactobacillus*, *Staphylococcus*, *Peptococcus, Marvinbryantia* and *Acidaminobacter* (−)		
Okra seed oil	Hepatoprotection	Ethanol-induced mice	400 and 800 mg/kg, 8 weeks	Phylum level: Bacteroidetes (+); Proteobacteria and Firmicutes/Bacteroidetes ratio (−)	NA	(92)
				Genus level: *Lactobacilli* (+)		
Hawthorn seed oil	Hypocholesterolemic effect	High-cholesterol diet-induced hamsters	4.75 and 9.5%, 6 weeks	Genus level: *Faecalibaculum*, *Ruminococcus_2* and *norank_f__Clostridiales_vadinBB60_group* (+); *unclassifed_f_Christensenellaceae*, *Ruminococcaceae_NK4A214_group*, *norank_o_Gastranaerophilales* and *Peptococcus* (−)	NA	(96)
Wild melon seeds oil	Hypocholesterolemic effect	High-cholesterol diet-induced hamsters	4.75 and 9.5%, 6 weeks	Phylum level: Bacteroidetes (+); Firmicutes (−)	NA	(93)
				Genus level: *Bifidobacterium*, *Ruminococcus_2*, *nonrank_f_Eubacteriaceae* and *unclassified_p_Firmicutes* (+); *Bilophila*, *Blautia*, *Lachnoclostridium*, *Lachnospiraceae_UCG_006*, *Ruminiclostridium_9*, *[Eubacterium]_coprostanoligenes_group* and *norank_f_Ruminococcaceae* (−)		
Tomato seed oil	Anti-hyperlipidemia	High-fat diet-induced mice	5.9 and 11.8%, 12 weeks	Phylum level: Bacteroidetes (+); Firmicutes and Firmicutes/Bacteroidetes ratio (−)	*norank_o__Gastranaerophilale*, *Phascolarctobacterium* and *Lactobacillus*	(94)
				Genus level: *Alistipes*, *Phascolarctobacterium*, *Lactobacillus*, and *Anaerotruncus* (+); *Rikenella* (−)		
Almond oil	Anti-diabetes	Streptozotocin-induced rats	2, 4 and 8 g/kg BW, 4 weeks	Phylum level: Bacteroidetes (+); Firmicutes (−)	NA	(95)
				Genus level: *Bacteroides*, *Lactobacillus* and *Lachnospiraceae_NK4A136_group* (+); *Ruminococcaceae_UCG-014*, *Clostridium_sensu_stricto_1* and *Fusicatenibacter* (−)		

Regarding soybean oil, it raised the abundances of Bacteroidetes, Deferribacteres, and Proteobacteria, and shrunk those of Firmicutes, Tenericutes, and TM7 at the phylum level while revealed anti-atherosclerosis action to normal mice (66). Moreover, 11 kinds of bacteria (*Prevotella*, *unclassified S24-7*, *Mucispirillum*, *Dehalobacterium*, *Ruminococcus*, etc.) were identified as the differential gut microbiota biomarkers. On the other hand, the amounts of six bacteria (*Akkermansia muciniphila*, *Turicibacter*, *Thermicanus*, *Clostridium saccharogumia*, *Lactobacillus pontis*, etc.) were upregulated, and that of *Coprobacillus* was downregulated, while soybean oil exhibited attenuate metabolic syndrome effect (67) against high-fat diet-induced mice and increase insulin sensitivity and prevent fatty liver activities in normal mice (68). To sea buckthorn seed/pulp oil, it increased the number of Bacteroidetes and decreased that of Firmicutes as well as Firmicutes/Bacteroidetes ratio at the phylum level, while exhibited hypocholesterolemic effect on high-cholesterol diet-induced hamsters (69). At the genus level, it enhanced the amounts of nine bacteria (*norank_f_Bacteroidales_S24-7_group*, *Allobaculum*, *norank_o_Mollicutes_RF9*, *Alistipes*, *Bacteroides*, etc.), and lowered those of 13 bacteria (*Acietatifactor*, *Lactobacillus*, *norank_f_Ruminococcaceae*, *Mucispirillum*, *Anaeroplasma*, etc.), while exerted hypocholesterolemic (69) and immunoenhancement (70) effects. Moreover, Tannerellaceae and *Parabacteroides* were characterized as differential gut microbiota biomarkers while sea buckthorn pulp oil exhibited immunoenhancement effect toward cyclophosphamide-induced mice (70). Otherwise, sea buckthorn seed and pulp oil enlarged the abundances of *Oscillibacter* and *Roseburia*, and shrunk that of *Lactobacillus* while exerted ameliorate non-alcoholic fatty liver disease effects in high-fat diet-induced mice (71).

Coconut oil could regulate gut microbiota during exerted improvements in diabetes (72), improving the gastrointestinal tract (73), anti-obese (17, 74, 75), and regulation of lipid metabolism (76) effects. At the phylum level, coconut oil increased the abundances of Firmicutes, Actinobacteria, and/or Bacteroidetes along with Firmicutes*/*Bacteroidetes ratio, and decreased those of Bacteroidetes, Spirochaetes, and/or Firmicutes (72, 74, 76). At the genus level, the amounts of *Lactobacillus*, *Allobaculum*, and *Erysipelotrichaceae_UCG-006* were augmented, and those of *Treponema* and *Turicibacter* were declined, while coconut oil reflected improved diabetes (72) and regulation of lipid metabolism (76) activities. On the other hand, coconut oil upregulated the numbers of six kinds of bacteria (*Alloprevotella*, Bifidobacteriales, *Lactobacilli*, Enterobacteriaceae, *Escherichia*, etc.), and downregulated those of *Corynebacterium*, Pseudomonadales, *Psychrobacter*, *Mitsuokella*, and *E. coli*, while exhibited improving gastrointestinal tract (73) and anti-obese (17, 75) activities.

Perilla oil, respectively, increased the abundances of Clostridiaceae and Lachnospiraceae while exhibited relieved constipation toward sedentary healthy female (21) and exerted improved gut function to athletes (77) at the family level. At the phylum level, the amounts of Bacteroidetes, Spirochaetes, and Verrucomicrobia were raised, and those of Firmicutes and Proteobacteria as well as Firmicutes*/*Bacteroidetes ratio were lessened, while perilla oil exerted anti-non-alcoholic fatty liver disease (78) and improved gut function (77) actions. At the genus level, the numbers of eight bacteria (*Alistipes*, *Alloprevotella*, *Parabacteroides*, *Rikenella*, *Roseburia*, etc.) were enhanced, while those of 11 bacteria (*unidentified_Ruminococcaceae*, *Lachnoclostridium*, *unidentified_Lachnospiraceae*, *Oscillibacter*, *Blautia*, etc.) were lowered, as perilla oil displayed anti-diabetes (79) and anti-non-alcoholic fatty liver disease (78) activities.

Peony seed oil declined the amounts of Proteobacteria and Deferribacteres accompanied by Firmicutes/Bacteroidetes ratio at the phylum level, while exhibited hypocholesterolemic (80) and alleviated hyperlipidemia and hyperglycemia (81) effects. At the genus level, peony seed oil increased the abundances of *unclassified_f_Ruminococcaceae*, *Ruminococcus_2*, *Lactobacillus*, *Prevotella* and/or *Parabacteroides*, and decreased those of 10 bacteria (*unclassified_f_Erysipelotrichaceae*, *Peptococcus*, *Christensenellaceae_R-7_group*, *norank_o_Mollicutes_RF9*, *norank_f_Eubacteriaceae*, etc.) (80, 81). Moreover, *Bacteroides*, *Turicibacter*, and *Allobaculum* were screened to be differential gut microbiota biomarkers, while peony seed oil alleviated hyperlipidemia and hyperglycemia against high-fat diet-induced mice (81). *Decaisnea insignis* seed oil enhanced the amounts of Saccharibacteria and/or Bacteroidetes and declined those of Firmicutes and Proteobacteria at phylum level, while showed hepatoprotection against alcohol or L-carnitine-induced mice (82, 83). At the genus level, the abundances of eight bacteria (*Lactobacillus*, *Conexibacter*, *Pepeococcus*, *Ruminoccoceae_UCG_004*, *Akkermansia*, etc.) were elevated, while those of *Helicobacter* and *Erysipelotrichaceae* were lowered (82, 83). Moreover, *Lactobacillus_gasseri* was identified to be differential gut microbiota biomarker for *Decaisnea insignis* seed oil that exhibited hepatoprotection on alcohol-induced mice (82). Sacha inchi oil increased the amount of Firmicutes, and decreased that of Bacteroidetes at the phylum level, while exerted hypolipidemic effect in high-fat diet-induced rats (84). At the genus level, it enhanced the abundance of *Alistipes*, and shrunk those of *Unidentified Enterobacteriaceae*, *Bacteroides*, and *Lachnoclostridium* (84). Moreover, six bacteria (*Roseburia*, *Turicibacter*, *Butyrivibrio*, *Unidentified Enterobacteriaceae*, *Escherichia*, etc.) were chosen to be differential gut microbiota biomarkers. While, sacha inchi oil upregulated the number of Bacteroidetes, and downregulated that of Firmicutes as well as Firmicutes/Bacteroidetes ratio at the phylum level, as exhibited regulation of lipid metabolism effect against high-fat diet-induced mice (85). Moreover, Deferribacteraceae, Deferribacterales, and Deferribacteres were identified to be differential gut microbiota biomarkers. Millet/riceberry bran oil increased the amounts of Verrucomicrobia, Saccharibacteria, and Bacteroidetes, and decreased that of Firmicutes along with Firmicutes/Bacteroidetes ratio at the phylum level, while exhibited attenuate metabolic syndrome effect (86) or anti-cancer activity (87). At genus level, the numbers of six bacteria (*Ruminococcaceae UCG-013*, *Ruminococcaceae UCG-014*, *Adlercreutzia*, *Enterorhabdus*, *Papillibacter*, etc.) were upregulated, and those of *Eubacterium coprostanoligenes*, *Ruminoclostridium 6* and *Bacteroides* were downregulated, while riceberry bran oil exerted anti-cancer action on diethylnitrosamine and 1,2-dimethylhydrazine-induced rats (87). Moreover, eight bacteria (*Akkernansia*, *unclassified_f__Lachnospiraceae, Prevotellaceae UCG_001*, *Erysipelatoclostridium*, *Ruminococcaceae UCG_009*, etc.) were screened to be differential gut microbiota biomarkers, as millet bran oil showed attenuate metabolic syndrome effect (86). *Torreya grandis* oil augmented the abundances of Firmicutes and Actinobacteria along with Firmicutes/Bacteroidetes ratio, and declined those of Bacteroidetes, Proteobacteria, and Verrucomicrobiota at the phylum level, while exhibited anti-obese (88) and anti-Alzheimer’s disease (89) effects. At the family level, *Torreya grandis* oil aggrandized the amounts of Erysipelotrichaceae, Coriobacteriaceae, Lactobacillaceae, and Bifidobacteriaceae, and reduced that of Porphyromonadaceae, while exerted anti-obese effect on high-fat diet-induced mice (88). At the species level, *Torreya grandis* oil raised the numbers of eight bacteria (*Allobaculum*, *Bifidobacterium*, *Olsenella*, *Parasutterella*, *unclassified Ruminococcaceae*, etc.), while showed anti-Alzheimer’s disease action against scopolamine-induced mice (89).

*Schizochytrium* sp. L. oil (85), safflower oil (90), peanut oil (43), kiwifruit seed oil (91), okra seed oil (92), wild melon seed oil (93), tomato seed oil (94) or almond oil (95) could also modulate gut microbiota during their health-promoting effects. At the phylum level, the abundances of Bacteroidetes and/or Deferribacteres as well as Firmicutes/Bacteroidetes ratio were increased, and those of Firmicutes, Bacteroidetes, Proteobacteria, and/or Actinobacteria along with Firmicutes/Bacteroidetes ratio were decreased (43, 85, 90–95). At the genus level, the abundances of 33 bacteria (*Blautia*, *Olsenella, Peptoclostridium, Ruminococcaceae_UCG-009, [Eubacterium]_fissicatena_group*, etc.) were enhanced, while these oils displayed the health-promoting effects (43, 90–96). On the contrary, those of 39 bacteria (*Barnesiella*, *Bilophila, Leuconostoc, [Eubacterium]_nodatum_group*, *Lactococcus,* etc.) were reduced. Moreover, for *Schizochytrium* sp. L. oil exerted regulation of lipid metabolism effect on high-fat diet-induced mice, Rhodospirillales and Alphaproteobacteria were identified to be differential gut microbiota biomarkers (85). In terms of peanut oil exhibited attenuate metabolic syndrome action against high-fat/high sucrose diet-induced rats, 10 types of bacteria (*g__Faecalibaculum*, *s__uncultured_bacterium_g_Faecalibaculum*, p__Proteobacteria, f_Clostridiaceae_1, *g__Ruminococcaceae_UCG_014*, etc.) were screened to be differential gut microbiota biomarkers (43). To kiwifruit seed oil showed anti-obese action on high-fat diet-induced mice, *Pseudoflavonifractor*, *Flavonifractor*, *Intestinimonas*, *Romboutsia* and *Olsenlla* were characterized to be differential gut microbiota biomarkers (91). Regarding tomato seed oil showed anti-hyperlipidemia effect against high-fat diet-induced mice, *norank_o_Gastranaerophilale*, *Phascolarctobacterium*, and *Lactobacillus* were chosen to be differential gut microbiota biomarkers (94).

Overall, there were other oils which could modulate gut microbiota during their health-promoting effects. Moreover, differential gut microbiota biomarkers have been acquired for some of them to show health-promoting effects.

### 3.5 Influences of dosage and treatment time of edible plant oils

Different dosages of camellia oil, olive oil, and flaxseed oil have been demonstrated to exhibit different behaviors in modulating gut microbiota during their health-promoting effects (16, 51, 56, 63, 68). In terms of camellia oil, 2 and 4 mL/kg BW of it significantly enriched the abundances of *Faecalibacterium*, *Muribaculaceae*, and *Coriobacteriaceae_UCG_002* in exercise mice, while 6 mL/kg BW of it obviously enhanced the amounts of *Desulfobacteria* and *Faecalibaculum* (16). Regarding olive oil, *Akkermansia muciniphila* was present at the dose of 7%, whereas *Mucispirillum schaedleri* was the most abundant at that of 21%, as it increased insulin sensitivity and prevented fatty liver in normal mice (68). For flaxseed oil, 400 mg/kg BW of it increased the quantities of *Lactobacillus* and *Lachnospiraceae_NK4A136_group*, while 1,600 mg/kg BW of it added the number of *Prevotellaceae_UCG-001* during its anti-ulcerative colitis effect in dextran sulfate sodium-induced rats (63).

Moreover, peony seed oil (80, 81), perilla oil (77, 79), *Torreya grandis* oil (88, 89), and other oils (69, 91, 93–95) have also been proven to generate different modulations on gut microbiota because of different doses during their health-promoting effects. For example, 1.5 mL of peony seed oil upregulated the proportions of *Lactobacillus* and *Prevotella*, while 1.0 mL of it elevated the presence of *Parabacteroides* and reduced the prevalence of *Ruminococcaceae_Ruminococcu* and *Mucispirillum*, as alleviated hyperlipidemia and hyperglycemia in high-fat diet-induced mice (81). For perilla oil exhibited anti-diabetes activity against high-fat diet-induced KKAy mice, *Dubosiella* and *Turicibacter* were the most abundant genus in low-dose (0.67 g/kg BW) group relative to middle-dose (1.33 g/kg BW) and high-dose (2.00 g/kg BW) groups, while *Lactobacillus* was the most abundant genus in the high-dose group compared to the low-dose and middle-dose groups (79). 250, 550, and 850 mg/kg of *Torreya grandis* oil decreased Porphyromonadaceae to 15.57, 10.96, and 6.97%, increased Erysipelotrichaceae to 20.32, 36.55, and 38.75%, and enhanced Coriobacteriaceae to 7.65, 1.94, and 3.07% during their anti-obese effects in high-fat diet-induced mice (88). 50% of sea buckthorn seed oil boosted the number of *norank_f_Bacteroidales_S24-7_group*, whereas 100% of it increased the amounts of *Allobaculum* and *norank_o_Mollicutes_RF9* in high-cholesterol diet-induced hamsters (69). 5.9% of tomato seed oil significantly changed the abundances of *Anaerotruncus* and *Alistipes*, while 11.8% of it obviously altered the amounts of *Lactobacillus*, *Rikenella*, and *Enterorhabdus* in high-fat diet-induced mice (94). The abundances of *Pseudoflavonifractor*, *Flavonifractor*, *Intestinimonas*, *Romboutsia*, and *Olsenlla* were markedly increased in the high-dose (3.0 mL/kg) kiwifruit seed oil group as compared to the low-dose (1.0 mL/kg) group, in high-fat diet-induced mice (91). 8 g/kg BW of almond oil notably changed the amounts of *Ruminococcaceae_UCG-014*, *Lactobacillus*, and *Lachnospiraceae_NK4A136_group*, 4 g/kg BW of it significantly altered the quantity of *Bacteroides*, and 2 g/kg BW of it obviously changed the abundances of *Clostridium_sensu_stricto_1* and *Fusicatenibacter* in streptozotocin-induced rats (95). 9.5% of wild melon seed oil showed significant differences in *Clostridiales_vadinBB60_group* and Streptococcaceae in comparison with 4.75% of it during its hypocholesterolemic effect in high-cholesterol diet-induced hamsters (93).

On the other hand, treatment time has been indicated to influence the modulations of edible plant oils on gut microbiota in three animal studies (67, 90, 97). The study taken by Hidalgo et al. (97) has shown that 6 weeks of olive oil intervention could not significantly alter the gut microbiota of normal ICR mice, whereas 12 weeks of that showed significant changes. The investigation of Danneskiold-Samsøe et al. (90) has indicated that significant differences were found in the abundances of *Bilophila*, *Olsenella*, *Clostridium XIVa*, *Parasutterella*, and *Pseudoflavonifactor* in the cecum as well as *Allobaculum* and *Parasutterella* in the colon at different treatment time points (2, 5, 10, and 40 weeks) of safflower oil in normal C57BL/6 J mice. Another study by Patrone et al. (67) has revealed that lower proportions of *Thermicanus*, *Akkermansia muciniphila*, *Lactobacillus pontis*, *Eubacterium biforme*, *Turicibacter*, but higher *Proteus*, *Ruminococcus flavefaciens*, *Clostridium perfringen*, *Allobaculum* and Deltaproteobacteria of high-fat diet-induced mice were found in coconut oil group as compared with soybean oil group at 2 weeks. While at 8 weeks, *Lactobacillus reuteri*, *F16*, *Anaerofustis*, *Allobaculum*, and Deltaproteobacteria were significantly higher, whereas *Akkermansia muciniphila* was lower in coconut oil group compared to soybean oil group.

### 3.6 Comparisons between edible plant oils and edible animal oils

Comparisons between different edible plant oils on gut microbiota modulations have been made in some studies. Compared with camellia oil, different modulations of corn oil, olive oil, soybean oil, perilla oil, and sunflower oil on the gut microbiota of mice or rats were seen while exhibited hepatoprotection, anti-ulcerative colitis, anti-obese, and anti-Alzheimer’s disease effects (31, 32, 36, 38, 42). For example, Lee et al. (32) have found that Prevotella was higher and Actinobacteria, Bacteroidetes, Firmicutes, and Proteobacteria were lower in camellia oil-treated ulcerative rats compared to soybean oil-treated ulcerative rats. Meanwhile, Actinobacteria and Firmicutes were higher and Bacteroidetes and Proteobacteria were lower in camellia oil-treated ulcerative rats compared to olive oil-treated ulcerative rats. Relative to olive oil, distinctions in gut microbiota modulation of mice, rats, or grouper have also been discovered in soybean oil, coconut oil, corn oil, and peanut oil as exerted increase insulin sensitivity and prevent fatty liver, attenuate metabolic syndrome, and improve gut health activities (43, 68, 98). For instance, as exhibited increase insulin sensitivity and prevent fatty liver effect on normal mice, 7% soybean oil treatment displayed a significant increase in *Akkermansia muciniphila*, whereas 7% olive oil consumption showed significant increments in 10 species (primarily *Bacteroides acidifaciens*, and *Faecalibacterium prausnitzii*), and 7% coconut oil administration had significant enhancements in eight species (particularly *Mucispirillum schaedleri*) (68). In comparison with flaxseed oil, different gut microbiota modulations of corn oil, olive oil, and soybean oil were generated in pigs, rats, and mice during their hepatoprotection, immunoregulation, anti-diabetes, and improving intrauterine growth retardation actions (50, 55, 59, 62). For example, as exerted anti-diabetes activity against streptozotocin-nicotinamide-induced rats, *Blautia* was higher and *Alistipes* was lower in corn oil group, as compared to flaxseed oil group (55). Additionally, distinctions of gut microbiota modulation to mice or suckling calves were found among soybean oil, peanut oil, coconut oil, palm oil, *Schizochytrium* sp. L. oil, sacha inchi oil, sea buckthorn pulp oil, sea buckthorn seed oil, walnut oil, and/or sunflower oil (65, 67, 71, 76, 85). For instance, significant differences of *Lactobacillus*, *Oscillibacter*, and/or *Roseburia* were observed among soybean oil, peanut oil, sea buckthorn seed oil, and sea buckthorn pulp oil treatments, during their ameliorate non-alcoholic fatty liver disease actions (71).

On the other hand, some studies have compared the gut microbiota modulations between edible plant oils and edible animal oils (including fish oil and lard oil) *in vivo* experiments (7, 57, 71, 78, 85, 98–100). In terms of comparisons with fish oil, the differential bacteria of *Schizochytrium* sp. L. oil group were Rhodospirillales and Alphaproteobacteria, whereas those of sacha inchi oil group were Deferribacteraceae, Deferribacterales, and Deferribacteres in high-fat diet-induced mice (85). Perilla oil has been reported to have similar gut microbiota modulations with fish oil in high-fat diet-induced rats (78). However, flaxseed oil was weaker than fish oil in modulating the gut microbiota of TMAO-induced *ApoE*^−*/*−^ mice (57). And, soybean oil, fish oil, and lard oil showed differential influences on the gut microbiota of middle-aged rats (100). Corn oil, olive oil, and fish oil had significantly different modulations on the amounts of *Photobacterium*, *Romboutsia*, *Epulopiscium*, *Clostridium_sensu_stricto_1*, *Staphylococcus* and/or *Leuconostoc* in grouper (98). Compared with lard oil, significant changes in *Blautia* were produced by peanut oil, sea buckthorn seed oil, and sea buckthorn pulp oil, in high-fat diet-induced mice (71). Soybean oil, olive oil, and lard oil treatments exhibited obviously different modulations on the gut microbiota of mice at phylum, class, order, and family levels (7). Corn oil and lard oil revealed notably different alterations in the gut microbiota of hamsters at phylum, family, and genus levels (99).

## 4 Correlations between gut microbiota and biochemical indexes affected by edible plant oils

Significant correlations between modulated gut microbiota and changed biochemical indexes were found while edible plant oils exhibited health-promoting effects, as summarized in Table 5.

**Table 5:** Correlations between modulated gut microbiota and changed biochemical indexes by edible plant oils.

Oil	Health-promoting effect	Experimental model	Correlated gut microbiota	Correlated biochemical indexes	References
Camellia oil	Mild cognitive impairment protection	Aluminum chloride-induced rats	*Ruminococcaceae_UCG-014*, *Bacteroides pectinophilus_group*, *Blautia*, *Lachnoclostridium* and *Prevotellaceae_UCG-001*	IL-6, TNF-α, IL-1β, BUN, GOT, SOD and IL-4	(36)
Camellia oil	Anti-Alzheimer’s disease	Aluminum chloride-induced rats	*[Eubacterium]_coprostanoligenes_group*, *Romboutsia*, *Clostridia_UCG-014*, *[Eubacterium]_xylanophilum_group*, *Monoglobus*, *Lachnoclostridium*, *Lactobacillus*, *Corynebacterium*, *Blautia*, *Lachnospiraceae_ND3007_group*, *f_Butyricicoccaceae_Unclassified*, *f_ Lachnospiraceae_Unclassified*, *Bacteroides*, *[Bacteroides]_pectinophilus_group*, *Roseburia, UCG-005*, *Prevotellaceae_NK3B31_group*, Muribaculaceae, *Christensensenellaceae_R-7_group*, *f_Oscillospiraceae_Unclassified*, *f_Ruminococcaceae_Unclassified*, *Atopostipes*, *Jeotgalicoccis* and *Staphylococcus*	Iba1, p-p65, APP, BACE1, AB1-42, MDA, IL-6, escape_time_trial_12, escape_time_work, IL-1β, TNF-α, LC3B, Atg5, GPx, IL-4, IL-10, brain_weight, SOD, Beclin1, CAT, time_spent_in target_quadrant and HDL	(8)
Camellia oil	Hypolipidemic effect	High-fat diet-induced mice	*Alistipes* and *Aerococcus*	mTOR and RpS6KB1	(39)
Camellia oil	Anti-fatigue	Rotarod test and Treadmill test in mice	*Enterorhabdus*, *Parvibacter*, *Akkermansia*, *Dubosiella*, *Clostridia_UCG-014*, *Bifidobacterium*, *Ruminococcus*, *[Eubacterium]_coprostanoligenes_group*, *Ileibacterium*, *Allobaculum*, *Desulfovibrio*, Oscillospiraceae, *Lactobacillus*, Muribaculaceae, *Alloprevotella*, *UCG-010*, *Alistipes*, Erysipelotrichaceae and *Bacteroides*	Rotarod time, running time, BUN, glycogen, ATP, GSH-Px, CAT, MDA, Keap1, Nrf2, NQO1, HO-1, GCLC, GCLM, SOD, myostain, FNDC5, Myh7, Myh2, Tnni1, Myh1, Myh4, ZO-1, Claudin-1 and Occludin	(16)
Camellia oil	Hepatoprotection	Alcohol-induced liver injury in mice	*Christensenellaceae_R-7_group*, *Escherichia_shigella*, *Eubacterium_nodatum_group*, *Coriobacteriaceae_UCG-002*, *Parvibacter*, *norank_f_Eggerthellaceae*, *Eubacterium_brachy_group*, *Harryflintia*, *Staphylococcus*, *Enterococcus*, *NK4A214_group*, *Faecalibaculum*, *GCA-900066575*, *UCG-005*, *norank_f_norank_o_Rhodospirillales*, *A2*, *Alistipes*, *Erysipelatoclostridium*, *Blautia*, *Romboutsia*, *Parabacteroides*, *Eubacterium_fissicatena_group* and *norank_f_norank_o_Clostridia_UCG-014*	Liver TG, ALT, MDA, and GSH-Px	(42)
Olive oil	Attenuate metabolic syndrome	Normal mice	*Desulfovibrio*, *Ruminiclostridium*, *Fusicatenibacter*, *Parasutterella*, *Olivibacter*, *Marispirillum*, *Spiroplasma*, *Marinilabilia*, *Desulfotomaculum* and *Helicobacter*	Food intake, water intake, diuresis, body weight, systolic blood pressure, leptin, Insulin, triglycerides, T-CHO, and HDL/LDL	(45)
Olive oil	Attenuate metabolic syndrome	Normal mice	*Prevotella*, *Marvinbryantia*, *Desulfovibrio*, *Anaerophaga*, *Fusicatenibacter*, *Parasutterella*, *Eubacterium*, Erysipelotrichaceae, *Olivibacter*, *Marispirillum* and *Enterobacter*	Water intake, HDL/LDL, T-CHO, blood pressure, leptin, insulin, ghrelin, and diuresis	(44)
Olive oil	Anti-hypertension	Spontaneously hypertensive rats	*Clostridia XIVa*	Systolic blood pressure	(46)
Olive oil	Anti-diabetes	NOD/LtJ mice	Verrucomicrobia, Cyanobacteria, Firmicutes, *Bacteroides*, *Akkermansia*, *Intestinimonas*, *Lachnospira*, *Eubacterium_xylanophilum_group*, Muribaculaceae, *Alistipes*, *Lachnoclostridium*, *Ruminococcaceae_UCG_005* and Gastranaerophilales	Islet number, C-Peptide, Random blood glucose, Th1/Th2, serum_TNF-*α*, insulitis, AUC area of OGTT, colon_IL-6, and colon_TNF-α	(48)
Flaxseed oil	Ameliorating polycystic ovary syndrome	Letrozole-induced rats	*Lactobacillus*, *Allobaculum*, Actinobacteria, *Bacteroides*, *Butyrivibrio*, *Desulfovibrio*, *Ruminiclostridium* and *Bifidobacterium*	LPS, FSH, FSH/LH, E2, PROG, T, SHBG, IL-1*β*, IL-6, IL-10, IL-17A, TNF-α, and MCP-1	(61)
Flaxseed oil	Anti-hepatocellular carcinoma	Orthotopic hepatocellular carcinoma mice	*Escherichia-shigella*, *Enterorhabdus*, *norank_f_norank_o_Clostridia UCG-014*, *Turicibacter, norank_f_norank_Clostridia_vadinBB60_group*, *Ruminococcus*, *Monoglobus*, *Eubacterium_brachy_group*, *unclassified_c_Bacteroidia* and *Lactococcus*	CD4*_*Tcells, CD8*_*Tcells, CD8*_*PD1*_*Tcells, Treges, TNF-α, IFN-γ, IL-10, TGF-β1, ZO-1, and Claudin-4	(18)
Flaxseed oil	Immunoregulation	Normal mice	Bacteroidetes	Triglyceride	(50)
Flaxseed oil	Anti-diabetes	Streptozotocin-nicotinamide-induced rats	Firmicutes, Bacteroidetes, *Blautia* and *Alistipes*	LPS, IL-1β, TNF-α, IL-6, and IL-17A	(55)
Flaxseed oil	Anti-atherosclerosis	High-fat diet-induced *ApoE*^−/−^ mice	*Intestinimonas*, *Alistipes*, *Muribaculum*, *Candidatus_Saccharimonas*, *Oscillibacter*, *Blautia*, *Parasutterella*, *Lachnoclostridium*, *Bilophila*, *Enterorhabdus*, *Anaerotruncus* and *Negativibacillus*	LPS, IL-1β, TNF-α, IL-6, IL-10, IL-17A, and MCP-1	(58)
Flaxseed oil	Regulation of lipid metabolism	Albas cashmere goats	*[Ruminococcus]_gauvreauii_group*, *Streptococcus*, *Turicibacter*, *Acetitomaculum*, *Bifidobacterium*, *Christensenellaceae_R-7_group*, *Mycoplasma*, *Olsenella*, *Ruminococcaceae_UCG-014*, *Ruminococcus_2*, *Ureaplasma*, *Lactobacillus*, *Lachnospiraceae_NK3A20_group*, *Intestinibacter*, *Family_XIII_AD3011_group*, *Clostridium_sensu_stricto_1*, *unclassified_f__Bifidobacteriaceae*, *[Eubacterium]_coprostanoligenes_group* and *Aeriscardovia*	Final body weight, total body weight gain, omental fat, kidney fat, mesenteric fat, glucose, β-hydroxybutyric acid, non-esterified fatty acid, triglyceride, cholesterol, low-density lipoprotein cholesterol, high-density lipoprotein cholesterol, acetyl-CoA carboxylase, fatty acid synthetase, hormone-sensitive lipase, lipoprotein lipase, and malic dehydrogenase	(52)
Flaxseed oil	Anti-aging	D-galactose-induced rats	*uncultured_bacterium_f_Muribaculaceae*, *Bacteroides*, *uncultured_bacterium_f_Desulfovibrionaceae*, *Ruminococcaceae_UCG-014*, *[Eubacterium]_oxidoreducens_group* and *Christensenellaceae_R-7_group*	GSH-Px, T-AOC, CAT, Prkcq, Lat, Ptprc, Lcp2, Lck, zap70, Pik3cd, and Nfatc2	(60)
Soybean oil	Increase insulin sensitivity and prevent fatty liver	Normal mice	*Ruminococcus bromii*, *Ruminococcus flavefaciens*, *Clostridium cocleatum*, *Bacteroides acidifaciens*, *Lactobacillus reuteri* and *Akkermansia muciniphila*	AUC, p-IRS, p-AKT, and LPS	(68)
Soybean oil	Attenuate metabolic syndrome	High-fat diet-induced mice	*Anaerotruncus*, *Lactobacillus_plantarum*, *Syntrophomonas*, *Akkermansia_muciniphila*, *Clostridium_saccharogumia*, *Staphylococcus_aureus*, *YS2*, *RF32*, *Allobaculum*, *Anaerofustis*, *F16*, Enterococcaceae, Actinomycetaceae and *Agrobacterium*	Crypt*_*depth, triglycerides, body weight, total and daily weight gain, mucosal lesion, leucocyte infiltration, Lep, ovarian fat, and cholesterol	(67)
Soybean oil	Anti-atherosclerosis	Normal mice	*unclassified Bacteroidales*, *Prevotella*, *unclassified S24.7, Mucispirillum*, *Dehalobacterium*, *Ruminococcus*, *unclassified Lachnospiraceae*, *unclassified Peptococcaceae*, *Coprobacillus*, *Sutterella*, *Bilophila*, *Desulfovibrio* and *Anaeroplasma*	Triglycerides, cholesterol, linoleic acid, lipid peroxides, and ROS	(66)
Walnut oil	Anti-Alzheimer’s disease	Scopolamine-induced mice	Firmicutes, Bacteroidetes, Proteobacteria, Cyanobacteria, and Verrucomicrobia	Slc6a4, Gng13, Kcnj12, Pik3r3, Kcnk3, Tph2, Kcnj4, Kcnq5, Plcb1, Cacnb1, Rgs2, Mylk3, Adcy5, Pde8b, Gng7, Chrm4, Htr2a, Kcnk2, Oxt, Gng10, Cacna1h, Kcna4, Htr6, Htr4, Agtr1a, Kcnq3, Chrm3, Cacng3, Camk2a, Cacnb3, Cacng8, Alox12b, Htr3a and Ptgs2	(64)
Peony seed oil	Alleviate hyperlipidemia and hyperglycemia	High-fat diet-induced mice	*Lactobacillus*, *Prevotellaceae_Prevotella*, *Bifidobacterium*, *Helicobacter*, *Prevotella*, *Parabacteroides*, *Coprococcus* and *Paraprevotella*	Glu, TC, TG, HDL, and LDL-C	(81)
Peony seed oil	Hypocholesterolemic effect	High-cholesterol diet-induced hamsters	*Anaeroplasma, Peptococcus, Butyricimonas, Candidatus_Saccharimonas, Christensenellaceae_R-7_group, norank_f_Clostridiales_vadinBB60_group*, *norank_f_Eubacteriaceae, norank_f_Lachnospiraceae, norank_f_Peptococcaceae, norank_o_Gastranaerophilales, norank_o_Mollicutes_RF9, Rikenellaceae_RC9_gut_group, Ruminiclostridium, Ruminiclostridium_1, Ruminiclostridium_9, Ruminococcaceae_NK4A214_group, unclassified_f_Coriobacteriaceae, unclassified_f_Christensenellaceae, unclassifed_f_Erysipelotrichaceae* and *unclassified_o_Bacteroidales*	TC, TG, LPS, protein_LXRa, SREBP2, HMG-CoA-R	(80)
Sea buckthorn seed oil	Hypocholesterolemic effect	High-cholesterol diet-induced hamsters	*Acietatifactor*, *Anaerovorax*, *Lactobacillus*, *[Eubacterium]_coprostanoligenes_group*, *unclassified_f_Ruminococcaceae*, *Parasutterella*, *Ruminococcaceae_UCG-014*, *norank_f_Rumincoccaceae*, *norank_f_Bacteroidales_S24-7_group* and *unclassified_k_norank*	TC, MTP, ACAT2, and ABCG8	(69)
Tomato seed oil	Anti-hyperlipidemia	High-fat diet-induced mice	*norank_f_Peptococcaceae*, *norank_f_Erysipelotrichaceae*, *norank_f_Clostridiales_vadinBB60_group*, *[Eubacterium]_fissicatena_group*, *Rikenella*, *Lactobacillus*, *Lachnoclostridium*, *Roseburia*, *Odoribacter*, *Faecalibaculum*, *Bifidobacterium* and *Allobaculum*	body weight, fat (perirenal + epididymal), TC, TG, LDL-C, HDL-C, and LDL-C/HDL-C	(94)
Almond oil	Anti-diabetes	Streptozotocin-induced rats	*Clostridium_sensu_stricto_1*, *Fusicatenibacter*, *Ruminococcaceae_UCG-014*, *Lachnospiraceae_NK4A136_group* and *Bacteroides*	FBG, insulin, body weight, SOD, CAT, MDA, TNF-α, IL-1β, Keap1, Nrf2, and HO-1	(95)
Wild melon seed oil	Hypocholesterolemic effect	High-cholesterol diet-induced hamsters	*Bilophila*, *Blautia*, *Escherichia-shigella*, *Lachnoclostridium*, *Lachnospiraceae_UCG-006*, *Lactococcus*, *Ruminiclostridium*, *Ruminiclostridium_9*, *Ruminococcaceae_UCG-004*, *[Eubacterium]_coprostanoligenes_group*, *norank_f_Eubacteriaceae, norank_f_Lachnospiraceae*, *norank_f_Ruminococcaceae*, *unclassified_f_Lachnospiraceae* and *unclassified_p_Firmicutes*	TC	(93)
*Torreya grandis* oil	Anti-obese	High-fat diet-induced mice	*Acetivibrio*, *Lactococcus*, *Eubacterium*, *Peptococcus*, *Clostridium*, *Sporobacter*, *Provencibacterium*, *Roseburia*, *Bacteroides*, *Lachnoclostridium*, *Akkermansia*, *Erysipelatoclostridium*, *Anaeroplasma*, *Lactobacillus*, *Faecalibaculum*, *Bifidobacterium*, *Allobaculum*, *Saccharofermentans*, *Paraeggerthella*, *Alistipes*, *Rikenella*, *Parabacteroides* and *Barnesiella*	TC, TG, HDL-C, and LDL-C	(88)
*Torreya grandis* oil	Anti-Alzheimer’s disease	Scopolamine-induced mice	*Anaeroplasma*, *Streptococcus*, *Allobaculum*, *Olsenella* and *Parasutterella*	Escape latency, percentage of spontaneity, DI, and SOD	(89)
Hawthorn seed oil	Hypocholesterolemic effect	High-cholesterol diet-induced hamsters	*Anaeroplasma*, *unclassified_f_Christensenellaceae, Candidatus_Saccharimonas, Peptococcus, norank_f_Peptococcaceae, Butyricimonas* and *norank_o_Gastranaerophilales*	TC and LPS	(96)
Safflower oil	Anti-obese	Obese people	*Adlercreutzia*, *Parabacteroides, Alistipes*, *Streptococcus*, *Anaerococcus*, *Blautia, Coprococcus, Hespellia, Parasporobacterium, Pseudobutyrivibrio, Roseburia, Faecalibacterium*, *Oscillibacter*, *Sporobacter*, Ruminococcaceae, Firmicutes and Co*probacillus*	TC, TG, LDL-C, and HDL-C	(20)

### 4.1 Affected by camellia oil

For camellia oil exerted mild cognitive impairment protection on aluminum chloride-induced rats (36), *Bacteroides pectinophilus_group* was positively correlated with TNF-*α*, IL-1β, and IL-6. *Blautia* was positively correlated with BUN, TNF-α, and IL-6. *Lachnoclostridium* and *Prevotellaceae_UCG-001* were positively correlated with GOT and TNF-α, respectively. *Ruminococcaceae_UCG-014* was negatively correlated with SOD and IL-4. For camellia oil exhibited anti-Alzheimer’s disease effect against aluminum chloride-induced rats (8), eight bacteria (*Romboutsia*, *Clostridia_UCG-014*, *[Eubacterium]_coprostanoligenes_group*, *[Eubacterium]_xylanophilum_group*, *Monoglobus*, etc.) had significantly positive correlations with 11 changed biochemical indexes (Iba1, p-p65, APP, BACE1, AB1-42, etc.), and had obviously negative relations with another 11 changed biochemical indexes (LC3B, Atg5, GPx, IL-4, IL-10, etc.). While 16 kinds of bacteria (*Blautia*, *Lachnospiraceae_ND3007_group*, *f_Butyricicoccaceae_Unclassified*, *f_ Lachnospiraceae_Unclassified*, *Bacteroides*, etc.) showed opposite correlations with these biochemical indexes.

Camellia oil revealed hypolipidemic effect on high-fat diet-induced mice (39), *Alistipes* was positively correlated with mTOR and RpS6KB1, and *Aerococcus* was negatively correlated with RpS6KB1. As this oil showed anti-fatigue action toward mice received with Rotarod test and Treadmill test (16), 19 bacteria (*Enterorhabdus*, *Parvibacter*, *Akkermansia*, *Dubosiella*, *Clostridia_UCG-014*, etc.) had significant correlations with 25 changed biochemical indexes (Rotarod time, Running time, BUN, glycogen, ATP, etc.) in serum, liver, muscle, and/or colon. Among them, Muribaculaceae was one of the differential gut microbiota biomarkers for camellia oil. Regarding this oil exhibited hepatoprotection against alcohol-induced liver injury in mice (42), *Enterococcus* and *NK4A214_group* were negatively correlated with liver TG, while *Faecalibaculum* was positively correlated with liver TG. *Staphylococcus*, *Enterococcus*, *Romboutsia*, and *Eubacterium_fissicatena_group* were negatively correlated with GSH-Px, whereas *Harryflintia* and *Parvibacter* were positively correlated with GSH-Px. *Alistipes* was negatively correlated with MDA, while *Eubacterium_brachy_group* and *Parvibacter* were positively correlated with MDA. *norank_f_Eggerthellaceae*, *Parvibacter*, and *Coriobacteriaceae_UCG-002* were negatively correlated with ALT, whereas 14 bacteria (*GCA-900066575*, *Staphylococcus*, *UCG-005*, *norank_f_norank_o_Rhodospirillales*, *Eubacterium_nodatum_group*, etc.) were positively correlated with ALT.

In a word, significant correlations between modulated gut microbiota and changed biochemical indexes occurred during camellia oil exhibited mild cognitive impairment protection, anti-Alzheimer’s disease, hypolipidemic, anti-fatigue, and hepatoprotection effects.

### 4.2 Affected by olive oil

For olive oil exerted attenuate metabolic syndrome effect on normal mice (45), *Desulfovibrio* was positively correlated with food intake, water intake, diuresis, and T-CHO. *Ruminiclostridium* was positively correlated with systolic blood pressure and HDL/LDL and was negatively correlated with water intake. *Fusicatenibacter* was positively correlated with water intake and diuresis and was negatively correlated with body weight, T-CHO, and HDL/LDL. *Parasutterella* was negatively correlated with insulin and HDL/LDL. *Olivibacter* was positively correlated with triglycerides and was negatively correlated with food intake. *Marispirillum* was negatively correlated with leptin, and *Spiroplasma* was negatively correlated with food intake and HDL/LDL. *Marinilabilia* was positively correlated with food intake, water intake, and diuresis. *Desulfotomaculum* was negatively correlated with food intake and T-CHO. *Helicobacter* was positively correlated with water intake and diuresis and was negatively correlated with leptin. In another study, as olive oil exhibited attenuate metabolic syndrome activity in normal mice (44), *Prevotella* was negatively correlated with T-CHO, while *Anaerophaga* was positively correlated with T-CHO. *Marvinbryantia* and *Eubacterium* were positively correlated with leptin, while *Marispirillum* was negatively correlated with leptin. *Desulfovibrio* was positively correlated with water intake, blood pressure, insulin, and diuresis. *Fusicatenibacter* was negatively correlated with HDL/LDL and T-CHO. *Parasutterella* was positively correlated with T-CHO and was negatively correlated with HDL/LDL. Erysipelotrichaceae was positively correlated with T-CHO and was negatively correlated with ghrelin. *Olivibacter* was positively correlated with T-CHO and ghrelin. *Enterobacter* was negatively correlated with water intake. Regarding olive oil showed anti-hypertension effect against spontaneously hypertensive rats, *Clostridia XIVa* was negatively correlated with systolic blood pressure (46). For olive oil revealed anti-diabetes action toward NOD/LtJ mice (48), *Lachnospira* and *Eubacterium_xylanophilum_group* were positively correlated with Random blood glucose, Th1/Th2, insulities, and AUC area of OGTT. Muribaculaceae was negatively correlated with serum_TNF-*α*. Verrucomicrobia and *Akkermansia* were negatively correlated with colon_TNF-α. Cyanobacteria and Gastranaerophilales were negatively correlated with insulitis, AUC area of OGTT, and colon_TNF-*α*. *Alistipes* was positively correlated with C-peptide and was negatively correlated with AUC area of OGTT. *Intestinimonas* was negatively correlated with insulitis. *Ruminococcaceae_UCG_005* was positively correlated with islet number and negatively correlated with Random blood glucose and AUC area of OGTT. *Lachnoclostridium* was negatively correlated with insulitis and AUC area of OGTT. *Bacteroides* was negatively correlated with colon_IL-6. Among them, Muribaculaceae, *Akkermansia*, Gastranaerophilales, *Alistipes*, *Intestinimonas*, *Ruminococcaceae_UCG_005*, Lachnoclostridium, and *Bacteroides* were differential gut microbiota biomarkers for olive oil.

Overall, notable correlations between modulated gut microbiota and changed biochemical indexes existed during olive oil exerted attenuate metabolic syndrome, anti-hypertension, and anti-diabetes actions.

### 4.3 Affected by flaxseed oil

For flaxseed oil exhibited ameliorating polycystic ovary syndrome effect on letrozole-induced rats (61), Firmicutes/Bacteroidetes ratio was positively correlated with E2, PROG, T, IL-1β, TNF-*α*, and MCP-1 in plasma and with IL-6, IL-17A, and MCP-1 in the ovary and was negatively correlated with IL-10 in the ovary. Actinobacteria was positively correlated with LPS, FSH/LH, E2, PROG, T, IL-6, and TNF-*α* in plasma and with IL-1β, IL-6, IL-17A, and TNF-α in the ovary and was negatively correlated with FSH and IL-10 in plasma. *Lactobacillus* was positively correlated with FSH and E2 in plasma and was negatively correlated with LPS, FSH/LH, T, and IL-6 in plasma and with IL-1β, TNF-*α*, and MCP-1 in ovary. *Bacteroides* was positively correlated with LPS, FSH/LH, IL-1β, IL-6, and TNF-α in plasma and with IL-1β, IL-6, IL-17A, and TNF-α in the ovary. *Allobaculum* was positively correlated with PROG in plasma and was negatively correlated with IL-17A and MCP-1 in plasma and with MCP-1 in ovary. *Butyrivibrio* was positively correlated with FSH, E2, PROG, SHBG, and IL-10 in plasma, and was negatively correlated with LPS, FSH/LH, T, IL-1β, IL-6, and MCP-1 in plasma and with IL-1β and MCP-1 in the ovary. *Desulfovibrio* was positively correlated with E2, PROG, SHBG, and IL-10 in plasma and with IL-10 in the ovary, and was negatively correlated with LPS, FSH/LH, T, IL-1β, IL-6, IL-17A, TNF-*α*, and MCP-1 in plasma and IL-17A, TNF-α and with MCP-1 in the ovary. *Ruminiclostridium* was positively correlated with FSH, PROG, and IL-10 in plasma, and was negatively correlated with T in plasma. *Bifidobacterium* was positively correlated with FSH, E2, and IL-10 in plasma and with IL-10 and IL-17A in the ovary, and was negatively correlated with LPS, FSH/LH, T, IL-1β, IL-6, TNF-*α*, and MCP-1 in plasma and with IL-6 in the ovary.

As flaxseed oil exerted anti-hepatocellular activity against orthotopic hepatocellular carcinoma mice (18), *Escherichia-shigella* was negatively correlated with CD8*_*Tcells and Claudin-4. *Enterorhabdus* was positively correlated with IFN-*γ* and was negatively correlated with CD8*_*PD1*_*Tcells and IL-10. *norank_f_norank_o_Clostridia UCG-014* was positively correlated with CD4*_*Tcells, CD8*_*Tcells and IFN-γ, and was negatively correlated with CD8*_*PD1*_*Tcells and IL-10. *Turicibacter* was positively correlated with TNF-*α* and was negatively correlated with IL-10. *norank_f_norank_Clostridia_vadinBB60_group* was positively correlated with CD4*_*Tcells, CD8*_*Tcells and IFN-γ, and was negatively correlated with CD8*_*PD1*_*Tcells and IL-10. *Ruminococcus* was positively correlated with CD4*_*Tcells and TNF-*α* and was negatively correlated with IL-10. *Monoglobus* was positively correlated with CD4*_*Tcells, CD8*_*Tcells, IFN-γ, and ZO-1, and was negatively correlated with CD8*_*PD1*_*Tcells and TGF-β1. *Eubacterium_brachy_group* was positively correlated with IL-10 and TGF-β1 and was negatively correlated with CD8*_*Tcells. *unclassified_c_Bacteroidia* was positively correlated with CD4*_*Tcells, CD8*_*Tcells and IFN-γ, and was negatively correlated with TGF-β1. *Lactococcus* was negatively correlated with ZO-1 and Claudin-4.

Regarding flaxseed oil showed immunoregulation in normal mice (50), Bacteroidetes was negatively correlated with plasma triglyceride concentration. For flaxseed oil revealed anti-diabetes effect toward streptozotocin-nicotinamide-induced rats (55), Firmicutes was positively correlated with LPS, IL-1β, TNF-*α*, IL-6, and IL-17A, Bacteroidetes was negatively correlated with LPS, IL-1β, TNF-α, and IL-17A, *Blautia* was positively correlated with LPS, IL-1β, TNF-α, and IL-6, and *Alistipes* was negatively correlated with LPS and TNF-α. As flaxseed oil displayed anti-atherosclerosis action against high-fat diet-induced *ApoE*^−/−^ mice (58), *Intestinimonas* was positively correlated with LPS and IL-1β in plasma and with IL-6 and IL-1β in the aorta. *Oscillibacter* was positively correlated with IL-17A in plasma and with TNF-α, IL-6, IL-1β, and IL-17A in the aorta, and was negatively correlated with IL-10. *Parasutterella* was positively correlated with IL-6. *Lachnoclostridium* was positively correlated with LPS in plasma and with TNF-α in the aorta. *Bilophila* was positively correlated with LPS and IL-1β in plasma and with TNF-α, IL-6, and IL-1β in the aorta. *Enterorhabdus* was positively correlated with TNF-α in the aorta. *Anaerotruncus* was positively correlated with IL-1β in plasma and with TNF-α in the aorta. *Negativibacillus* was positively correlated with LPS and IL-1β in plasma.

For flaxseed oil reflected regulation of lipid metabolism activity on Albas cashmere goats (52), 19 bacteria (*[Ruminococcus]_gauvreauii_group*, *Streptococcus*, *Turicibacter*, *Acetitomaculum*, *Bifidobacterium*, etc.) exhibited significant correlations with 17 changed biochemical indexes (final body weight, total body weight gain, omental fat, kidney fat, mesenteric fat, etc.).

As flaxseed oil exhibited anti-aging effect against D-galactose-induced rats (60), *uncultured_bacterium_f_Muribaculaceae* was negatively correlated with *Nfatc2*. *Christensenellaceae_R-7_group* was negatively correlated with *Prkcq*, *Ptprc*, *Lcp2*, *Lck*, *zap70*, and *Pik3cd*. *Bacteroide*s was positively correlated with T-AOC in serum, while *[Eubacterium]_oxidoreducens_group* was negatively correlated with T-AOC in serum. *uncultured_bacterium_f_Desulfovibrionaceae* was negatively correlated with GSH-Px in the liver. *Ruminococcaceae_UCG-014* was positively correlated with T-AOC in the liver, and *[Eubacterium]_oxidoreducens_group* was negatively correlated with T-AOC in the liver. *[Eubacterium]_oxidoreducens_group* was negatively correlated with CAT in the liver.

In short, obvious correlations between modulated gut microbiota and changed biochemical indexes appeared during flaxseed oil displayed ameliorating polycystic ovary syndrome, anti-hepatocellular, immunoregulation, anti-diabetes, anti-atherosclerosis, regulation of lipid metabolism, and anti-aging effect activities.

### 4.4 Affected by other oils

For soybean oil exhibited increase insulin sensitivity and prevented fatty liver effect on normal mice (68), *Ruminococcus bromii*, *Ruminococcus flavefaciens*, and *Clostridium cocleatum* were positively correlated with p-IRS and p-AKT, and were negatively correlated with AUC. *Bacteroides acidifaciens* was positively correlated with LPS, and *Lactobacillus reuteri* and *Akkermansia muciniphila* were negatively correlated with LPS. As soybean oil exerted attenuate metabolic syndrome action against high-fat diet-induced mice (67), *Anaerotruncus* was positively correlated with body weight and ovarian fat. *Lactobacillus_plantarum*, *Akkermansia_muciniphila*, and *Clostridium_saccharogumia* were positively correlated with Lep. *Syntrophomonas* was positively correlated with mucosal lesion, leucocyte infiltration, and Lep. *Staphylococcus aureus* was positively correlated with total and daily weight gain, body weight, and ovarian fat. *YS2* was positively correlated with cholesterol and ovarian fat and was negatively correlated with Crypt*_*depth. *RF32* and Enterococcaceae were negatively and positively correlated with Crypt*_*depth, respectively. *Allobaculum* was positively correlated with Lep and cholesterol. *Anaerofustis* was positively correlated with triglycerides and cholesterol; *F16* was positively correlated with cholesterol. Actinomycetaceae was negatively correlated with cholesterol and body weight, and *Agrobacterium* was negatively correlated with total and daily weight gain. Regarding soybean oil showed anti-atherosclerosis activity in normal mice (66), 13 bacteria (*unclassified Bacteroidales*, *Prevotella*, *unclassified S24.7, Mucispirillum*, *Dehalobacterium*, etc.) had significant correlations with triglycerides, cholesterol, linoleic acid, lipid peroxides and/or ROS in serum, peritoneal macrophages, or aorta. Among them, *Prevotella*, *unclassified S24.7, Mucispirillum*, *Dehalobacterium*, *Ruminococcus*, *unclassified Peptococcaceae*, *Coprobacillus*, *Bilophila*, *Desulfovibrio*, and *Anaeroplasma* were differential gut microbiota biomarkers for soybean oil.

For peony seed oil exhibited hypocholesterolemic effect against high-cholesterol diet-induced hamsters (80), eight bacteria (*Butyricimonas*, *norank_f_Peptococcaceae*, *norank_o_Gastranaerophilales*, *unclassified_f_Coriobacteriaceae*, *Peptococcus*, etc.) were positively correlated with TC, while *Anaeroplasma*, *Candidatus_Saccharimonas*, *Rikenellaceae_RC9_gut_group*, and *Ruminiclostridium_1* were negatively correlated with TC. Six bacteria (*norank_f_Eubacteriaceae*, *norank_o_Mollicutes_RF9*, *Candidatus_Saccharimonas*, *norank_f_Clostridiales_vadinBB60_group*, *Rikenellaceae_RC9_gut_group*, etc.) were negatively correlated with TG. Seven bacteria (*Butyricimonas*, *norank_f_Peptococcaceae*, *norank_o_Gastranaerophilales*, *Papillibacter*, *Peptococcus*, etc.) were positively correlated with LPS, while *Anaeroplasma* and *Candidatus_Saccharimonas* were negatively correlated with LPS. Six bacteria (*Christensenellaceae_R-7_group*, *norank_f_Lachnospiraceae*, *norank_o_Mollicutes_RF9*, *Ruminiclostridium*, *Ruminococcaceae_NK4A214_group*, etc.) were negatively correlated with protein_LXRa. *unclassifed_f_Erysipelotrichaceae* was positively correlated with SREBP2. *norank_f_Peptococcaceae*, *Peptococcus*, *Ruminococcaceae_NK4A214_group* and *unclassified_f_Christensenellaceae* were positively correlated with HMG-CoA-R. As sea buckthorn seed oil exerted hypocholesterolemic effect on high-cholesterol diet-induced hamsters (69), *Acietatifactor*, *Anaerovorax*, *Lactobacillus*, *[Eubacterium]_coprostanoligenes_group* and *unclassified_f_Ruminococcaceae* were positively correlated with TC, while *Parasutterella* and *Ruminococcaceae_UCG-014* were negatively correlated with TC. *Norank_f_Rumincoccaceae* and *unclassified_k_norank* were positively correlated with MTP, while *norank_f_Bacteroidales_S24-7_group* was negatively correlated with MTP. *Lactobacillus* was positively correlated with ACAT2, and *unclassified_k_norank* was positively correlated with ABCG8. Regarding hawthorn seed oil reflected hypocholesterolemic effect on high-cholesterol diet-induced hamsters (96), *unclassified_f_Christensenellaceae*, *Peptococcus*, *norank_f_Peptococcaceae*, *Butyricimona*, and *norank_o_Gastranaerophilales* were positively correlated with TC, and *Anaeroplasma* and *Candidatus_Saccharimonas* were negatively correlated with TC. *Unclassified_f_Christensenellaceae* and *norank_o_Gastranaerophilales* were positively correlated with LPS, while *Anaeroplasma* and *Candidatus_Saccharimonas* were negatively correlated with LPS. For wild melon seed oil reflected hypocholesterolemic effect toward high-cholesterol diet-induced hamsters (93), 13 bacteria (*Bilophila*, *Blautia*, *Escherichia-shigella*, *Lachnoclostridium*, *Lachnospiraceae_UCG-006*, etc.) were positively correlated with TC, whereas *norank_f_Eubacteriaceae* and *unclassified_p_Firmicutes* were negatively correlated with TC.

Regarding walnut oil displayed anti-Alzheimer’s disease effect toward scopolamine-induced mice (64), Firmicutes, Bacteroidetes, Proteobacteria, Cyanobacteria, and Verrucomicrobia had significant correlations with differential genes enriched in the cortisol synthesis and secretion pathway, oxytocin signaling pathway, cholinergic signaling pathway, and serotonergic signaling pathway. *Torreya grandis* oil revealed anti-Alzheimer’s disease action against scopolamine-induced mice (89), and *Anaeroplasma* was positively correlated with escape latency. *Streptococcus* was negatively correlated with percentage of spontaneity. *Allobaculum* and *Olsenella* were positively correlated with DI. *Parasutterella* was positively correlated with SOD.

For *Torreya grandis* oil showed anti-obese activity on high-fat diet-induced mice (88), 23 bacteria (*Acetivibrio*, *Lactococcus*, *Eubacterium*, *Peptococcus*, *Clostridium*, etc.) had significant correlations with serum TC, TG, HDL-C, and LDL-C. As safflower oil exhibited anti-obese effect on obese people (20), 17 types of bacteria (*Adlercreutzia*, *Parabacteroides, Alistipes*, *Streptococcus*, *Anaerococcus*, etc.) exhibited notable correlations with serum TC, TG, LDL-C, and HDL-C.

Regarding tomato seed oil showed anti-hyperlipidemia action against high-fat diet-induced mice (94), *Rikenella* and *Odoribacter* were positively correlated with body weight, while *Bifidobacterium* and *Allobaculum* were negatively correlated with body weight. *Odoribacter* was positively correlated with fat (perirenal + epididymal). *norank_f_Clostridiales_vadinBB60_group* and *Lactobacillus* were negatively correlated with TC. *Rikenella* was positively correlated with TG, while *norank_f_Clostridiales_vadinBB60_group* was negatively correlated with TG. *Bifidobacterium* was negatively correlated with LDL-C. *Roseburia*, *Lachnoclostridium*, and *Faecalibaculum* were negatively correlated with HDL-C. *Faecalibaculum* was positively correlated with LDL-C/HDL-C, whereas *Lactobacillus* was negatively correlated with LDL-C/HDL-C. Among them, *Lactobacillus* was one of the differential gut microbiota biomarkers for tomato seed oil. For peony seed oil reflected alleviated hyperlipidemia and hyperglycemia effect on high-fat diet-induced mice (81), *Bifidobacterium* was positively correlated with Glu, while *Prevotellaceae_Prevotella*, *Prevotella*, *Parabacteroides*, and *Paraprevotella* were negatively correlated with Glu. *Lactobacillus* and *Prevotella* were negatively correlated with TC. *Bifidobacterium* was positively correlated with TG, *Helicobacter* was positively correlated with HDL, and *Coprococcus* was positively correlated with LDL-C. As almond oil displayed anti-diabetes activity in streptozotocin-induced rats (95), *Clostridium_sensu_stricto_1* was positively correlated with FBG, MDA, TNF-*α*, IL-1β, and Keap1 and was negatively correlated with insulin, body weight, SOD, CAT, Nrf2, and HO-1. *Ruminococcaceae_UCG-014* was negatively correlated with body weight, SOD, Nrf2, and HO-1. *Fusicatenibacter* was positively correlated with FBG, MDA, TNF-α, IL-1β, and Keap1, and was negatively correlated with insulin, body weight, SOD, CAT, and Nrf2. *Lachnospiraceae_NK4A136_group* was positively correlated with SOD and Nrf2 and was negatively correlated with FBG and IL-1β. *Bacteroides* was positively correlated with insulin, SOD, CAT, Nrf2, and HO-1 and was negatively correlated with FBG and IL-1β.

Overall, remarkable correlations between modulated gut microbiota and changed biochemical indexes were also observed during other oils reflected increase insulin sensitivity and prevent fatty liver, attenuate metabolic syndrome, anti-atherosclerosis, hypocholesterolemic, anti-Alzheimer’s disease, anti-obese, anti-hyperlipidemia, alleviate hyperlipidemia and hyperglycemia, and anti-diabetes effects.

## 5 Gut microbiota-derived metabolites altered by edible plant oils

### 5.1 Alterations on SCFAs

Camellia oil increased the levels of propionic acid and butyric acid in feces while exerted Alzheimer’s disease effect on aluminum chloride-induced rats (8). Meanwhile, this oil elevated the amounts of total SCFAs, acetic acid, propionic acid, butyric acid, isobutyric acid, valeric acid, and isovaleric acid in colon contents, while exhibited anti-ulcerative colitis action against dextran sulfate sodium-induced mice (33). The oil reduced the contents of acetic acid, butyric acid, isobutyric acid, isovaleric acid and pentanoic acid in colonic contents, as showed hypolipidemic activity toward high-fat diet-induced mice (39).

Olive oil and flaxseed oil enhanced propionic acid production in plasma while displayed immunoregulation in normal mice (50). Moreover, Flaxseed oil supplementation increased the levels of acetic acid, propionic acid, butyric acid, isobutyric acid, valeric acid, and/or isovaleric acid in feces, while exhibited ameliorating polycystic ovary syndrome (61), anti-hepatocellular carcinoma (18), anti-atherosclerosis (57, 58) and anti-diabetes (55) effects.

Sea buckthorn pulp oil increased the concentrations of acetic acid, butyric acid, and total SCFAs in feces, while exerted immunoenhancement effect on cyclophosphamide-induced mice (70). Sea buckthorn seed oil elevated the levels of acetic acid, propionic acid, butyric acid, and total SCFAs in feces, as exhibited hypocholesterolemic effect against high-cholesterol diet-induced hamsters (69). Coconut oil enhanced the production of propionic acid and butyric acid in feces, while showed anti-obese effect on obese rats (75). Riceberry bran oil aggrandized the levels of butyric acid and valeric acid and reduced that of acetic acid in feces, while showed anti-cancer activity in diethylnitrosamine and 1,2-dimethylhydrazine-induced rats (87). Walnut oil raised the amounts of total SCFAs, acetic acid, propionic acid, and butyric acid in feces, as revealed anti-ulcerative colitis activity against dextran sulfate sodium-induced colitis mice (65). Walnut oil increased the levels of propionic acid, butyric acid, and valeric acid in plasma, while displayed anti-Alzheimer’s disease effect in scopolamine-induced mice (64). *Decaisnea insignis* seed oil enhanced the concentrations of total SCFAs, acetic acid, propionic acid, and butyric acid in feces, as revealed hepatoprotection in L-carnitine-induced mice (83). Wild melon seed oil increased the levels of total SCFAs, acetic acid, propionic acid, and butyric acid in feces, as showed hypocholesterolemic effect on high-cholesterol diet-induced hamsters (93). *Torreya grandis* oil elevated the butyric acid concentration in serum, while exerted anti-Alzheimer’s disease action against scopolamine-induced mice (89). Hawthorn seed oil added the contents of total SCFAs, acetic acid, propionic acid, and butyric acid in feces, as revealed hypocholesterolemic effect on high-cholesterol diet-induced hamsters (96). Okra seed oil decreased concentrations of acetic acid, isobutyric acid, and isovaleric acid, and increased that of butyric acid in cecal contents of ethanol-induced mice (92).

### 5.2 Alterations on other gut microbiota-derived metabolites

Flaxseed oil intervention modulated the microbial bile acids (BAs) metabolism in feces while exhibited anti-atherosclerosis action on high-fat diet-induced *ApoE*^−/−^ mice, showing increases of lithocholic acid, allocholic acid, glycocholic acid, and taurocholic acid and decreases of allolithocholic acid, isolithocholic acid, 7-ketodeoxycholic acid, *β*-ursodeoxycholic acid, chenodeoxycholic acid, and hyodeoxycholic acid (58). Flaxseed oil caused a higher conjugated BA concentration and conjugated/free BA ratio in cecal contents in high-fat diet-induced mice (51). Thereinto, the concentrations of several unconjugated BAs, including chenodeoxycholic acid and β-murocholic acid, were higher. Moreover, the concentrations of taurine-conjugated BAs, and in particular taurocholic acid and tauro-ursodesoxycholic acid were increased. Furthermore, the amounts of cholic acid and tauro-β-murocholic acid were elevated. Sacha inchi oil declined primary BAs including cholic acid, glycocholic acid, taurochenodeoxycholic acid, and taurocholic acid in the feces of high-fat diet-induced rats (84). Meanwhile, it significantly modulated the levels of glycerolipids and glycerophospholipids in the liver.

Sea buckthorn seed oil changed the compositions of hepatic fatty acids and fecal fatty acids of high-cholesterol diet-induced hamsters (69). Tomato seed oil reduced saturated fatty acids, monounsaturated fatty acids, and total fatty acid concentrations, and added polyunsaturated fatty acids in the liver and/or feces of high-fat diet-induced mice (94). Okra seed oil increased the levels of pentadecanoic acid, palmitic acid, heptadecanoic acid, linoleic acid, oleic acid, etc., and decreased those of myristic acid and *trans*-9-octadecenoic acid in serum, while showed hepatoprotection on ethanol-induced mice (92). Coconut oil changed the fatty acid composition of the liver and longissimus dorsi while exhibited regulation of lipid metabolism effect in Holstein male calves (76). *Torreya grandis* oil supplement generated alterations in fatty acid composition in livers of high-fat diet-induced mice, revealing increments of eicosadienoic acid, sciadonic acid, *n*-6 PUFA, MUFA, and PUFA, and reductions of arachidonic acid and SFA (88).

About 117 metabolites and 392 metabolites in feces were, respectively, upregulated and downregulated by camellia oil treatment, while it revealed gastroprotection on ethanol-induced mice (41). These metabolites were amino acid and its metabolites, terpenoids, heterocyclic compounds, and lignans compounds, which were significantly enriched in four key metabolic pathways, including biosynthesis of cofactors, metabolic pathway, purine metabolism, and ammonia acid biosynthesis. 159 significantly different metabolites (lipids and lipid-like molecules) in serum metabolites of mice were identified between olive oil-treated group and diabetic group (48). These metabolites were significantly enriched in 18 metabolic pathways. *Decaisnea insignis* seed oil led to increases in concentrations of squalene, hesperidin, 2-aminophenol, 5-hydroxyindole-3-acetic acid, abietic acid, etc., and decreases in levels of androsterone, triacetin, farnesal, indole-3-acetamide, thymidine-5′-monophosphate dreg pored, etc., in the cecal contents of alcohol-induced mice (82). Moreover, five correlated KEGG pathways were enriched by these metabolites, including steroid hormone biosynthesis, steroid biosynthesis, pyrimidine metabolism, tryptophan metabolism, and tryptophan metabolism pathways. Peony seed oil notably changed 30 metabolites in feces of high-fat diet-induced mice, including organic acids and derivatives, phenylpropanoids and polyketides, benzenoids, organoheterocyclic compounds, lipids and lipid-like molecules, etc. (81).

### 5.3 Correlations between altered metabolites and modulated gut microbiota

#### 5.3.1 SCFAs and gut microbiota

For camellia oil exhibited anti-Alzheimer’s disease effect on aluminum chloride-induced rats (8), the acetic acid in feces was positively correlated with *Prevotellaceae_NK3B31_group*, and negatively correlated with *f__Ruminococcaceae_Unclassified*, *Atopostipes*, *Jeotgalicoccus* and *Staphylococcus*. Meanwhile, the butyric acid in feces was positively correlated with *Clostridia_UCG-014* and *Lachnoclostridium*, and negatively correlated with *Blautia*, *Lachnospiraceae_ND3007_group*, *f__Butyricicoccaceae_Unclassified*, *UCG-005*, and *Christensenellaceae_R-7_group*. Additionally, the propionic acid in feces was negatively correlated with *Roseburia*.

Regarding flaxseed oil exerted anti-hepatocellular carcinoma effect against orthotopic hepatocellular carcinoma mice (18), the acetic acid in feces was negatively correlated with *Eubacterium brachy_group*. Meanwhile, the butyric acid, isobutyric acid, propionic acid, and isovaleric acid in feces were negatively correlated with *Lactococcus*. In addition, the valeric acid in feces was negatively correlated with *Eubacterium brachy_group*, *Lactococcus*, and *Parabacteroides*. As flaxseed oil exhibited anti-atherosclerosis action on TMAO-induced *ApoE*^−/−^ mice (57), the acetic acid in feces was positively correlated with *Alistipes*, *Bifidobacterium*, *Odoribacter*, *Parasutterella*, and *norank Bacteroidales S24-7*, and negatively correlated with seven bacteria (*Blautia*, *Clostridium_sensu_stricto_1*, *Lactococcus*, *Romboutsia*, *Roseburia*, etc.). The propionic acid in feces was positively correlated with six bacteria (*Alistipes*, *Bifidobacterium*, *Coriobacteriaceae UCG-002*, *Odoribacter*, *Parasutterella*, etc.), and negatively correlated with 15 bacteria (*Anaerotruncus*, *Bilophila*, *Blautia*, *Clostridium_sensu_stricto_1*, *Lachnoclostridium*, etc.). The butyric acid in feces was positively correlated with *Alistipes*, *Desulfovibrio*, *Lactobacillus*, and *Odoribacter*, and negatively correlated with six bacteria (*Akkermansia*, *Bilophila*, *Blautia*, *Coriobacteriaceae UCG-002*, *Faecalibaculum*, etc.). The valeric acid in feces was positively correlated with six bacteria (*Alistipes*, *Desulfovibrio*, *Enterorhabdus*, *Lactobacillus*, *Odoribacter*, etc.), and negatively correlated with *Akkermansia*, *Blautia*, *Clostridium_sensu_stricto_1*, *Faecalibaculum*, and *Roseburia*. The total SCFAs in feces were positively correlated with *Alistipes*, *Bifidobacterium*, *Odoribacter*, *Parasutterella*, and *norank Bacteroidales S24-7*, and negatively correlated with 12 bacteria (*Anaerotruncus*, *Blautia*, *Clostridium_sensu_stricto_1*, *Lachnoclostridium*, *Lactococcus*, etc.). On the other hand, for flaxseed oil revealed anti-atherosclerosis action on high-fat diet-induced *ApoE*^−/−^ mice (58), the acetic acid and propionic acid in feces were negatively correlated with seven bacteria (*Intestinimonas*, *Oscillibacter*, *Lachnoclostridium*, *Bilophila*, *Enterorhabdus*, etc.). The isobutyric acid in feces was negatively correlated with *Intestinimonas*, *Oscillibacter*, *Blautia*, and *Bilophila*. The isovaleric acid in feces was negatively correlated with *Intestinimonas*, *Blautia*, *Bilophila*, *Enterorhabdus*, and *Negativibacillus*. The valeric acid in feces was negatively correlated with eight bacteria (*Intestinimonas*, *Oscillibacter*, *Blautia*, *Lachnoclostridium*, *Bilophila*, etc.). For flaxseed oil displayed ameliorating polycystic ovary syndrome activity in letrozole-induced rats (61), the acetic acid in feces was positively correlated with *Lactobacillus*, *Butyrivibrio*, *Desulfovibrio*, and *Bifidobacterium*, and negatively correlated with Firmicutes*/*Bacteroidetes ratio. The propionic acid in feces was positively correlated with *Lactobacillus*, *Butyrivibrio*, and *Desulfovibrio*, and negatively correlated with Firmicutes*/*Bacteroidetes ratio. The butyric acid in feces was positively correlated with *Lactobacillus*, *Allobaculum*, *Butyrivibrio*, and *Desulfovibrio*, and negatively correlated with Actinobacteria and *Bacteroides*. The valeric acid in feces was positively correlated with *Butyrivibrio*, and negatively correlated with Actinobacteria and *Bacteroides*.

For sea buckthorn seed oil exerted hypocholesterolemic effect against high-cholesterol diet-induced hamsters (69), the acetic acid, propionic acid, valeric acid, and total SCFAs in feces were positively correlated with *Parasutterella* and *Ruminococcaceae_UCG-014*, and negatively correlated with *Acietatifactor*, *Lactobacillus*, and *[Eubacterium]_coprostanoligenes_group*. Meanwhile, the acetic acid was negatively correlated with *Anaerovorax* and *Butyricimonas*, the propionic acid was negatively correlated with *unclassified_f_Ruminococcaceae* and *Ruminococcaceae_UCG-009*, the valeric acid was negatively correlated with *unclassified_f_Ruminococcaceae*, *Coriobacteriaceae_UCG-002*, *Butyricimonas*, and *Anaerovorax*, and the total SCFAs was negatively correlated with *unclassified_f_Ruminococcaceae*, *Coriobacteriaceae_UCG-002*, and *Butyricimonas*. Moreover, the butyric acid in feces was positively correlated with *Parasutterella*, and negatively correlated with *unclassified_f_Ruminococcaceae*, *[Eubacterium]_coprostanoligenes_group*, *Lactobacillus*, *Butyricimonas*, and *Acietatifactor*. As tomato seed oil exhibited anti-hyperlipidemia activity in high-fat diet-induced mice (94), the acetic acid in feces was positively correlated with *Allobaculum*, *Bifidobacterium*, *norank_f_Erysipelotrichaceae* and *unclassified_f_Erysipelotrichaceae*, and negatively correlated with *Anaerotruncus*, *Lachnospiraceae_NK4A136_group*, *Odoribacter*, and *[Eubacterium]_fissicatena_group*. The propionic acid was positively correlated with *unclassified_f_Erysipelotrichaceae*, and negatively correlated with *Lactobacillus*, *norank_f_Clostridiales_vadinBB60_group*, and *norank_f_Peptococcaceae*. The butyric acid was positively correlated with *Ruminiclostridium_5*, and negatively correlated with *norank_o_Gastranaerophilales*. The valeric acid was positively correlated with *Bifidobacterium*, *Pseudomonas*, and *norank_f_Peptococcaceae*, and negatively correlated with *[Eubacterium]_fissicatena_group*. The total SCFAs were positively correlated with *Allobaculum*, *Bifidobacterium*, *norank_f_Erysipelotrichaceae*, and *unclassified_f_Erysipelotrichaceae*, and negatively correlated with *Anaerotruncus*. Among them, *Lactobacillus* and *norank_o_Gastranaerophilales* were two differential gut microbiota biomarkers for tomato seed oil. Regarding *Decaisnea insignis* seed oil displayed hepatoprotection on L-carnitine-induced mice (83), the acetic acid in feces was positively correlated with *Alistipes*. The propionic acid was negatively correlated with eight bacteria (*norank_f_Erysipelotrichaceae*, *Turicibacter*, *[Eubacterium]_fissicatena_group*, *Clostridium_sensu_stricto_1*, *Faecalibaculum*, etc.). The butyric acid was positively correlated with 13 bacteria (*unclassified_o_Bacteroidales*, *norank_o_Mollicutes_RF9*, *Ruminococcus_1*, *[Eubacterium]_xylanophilum_group*, *[Eubacterium]_fissicatena_group*, etc.), and negatively correlated with eight bacteria (*Alistipes*, *Lachnospiraceae_NK4A136_group*, *Odoribacter*, *Rikenella*, *Ruminiclostridium*, etc.). The isovaleric acid was negatively correlated with *norank_f_Ruminococcaceae*, *Helicobacter*, and *Clostridium_sensu_stricto_1*. The valeric acid was positively correlated with *Ruminiclostridium* and *Prevotellacee_NK3B31_group*. For wild melon seeds oil showed hypocholesterolemic effect against high-cholesterol diet-induced hamsters (93), the acetic acid, propionic acid, butyric acid, valeric acid, and total SCFAs in feces were positively correlated with *unclassified_p_Firmicutes*, *nonrank_f_Eubacteriaceae*, *norank_f__Clostridiales_vadinBB60_group* and *Ruminococcus_2*, and negatively correlated with *norank_f_Ruminococcaceae* and *Escherichia-shigella*. In addition, the acetic acid was positively correlated with *norank_o_Mollicutes_RF9*, *Rikenellaceae_RC9_gut_group* and *Bifidobacterium*, and negatively correlated with *norank_f_Lachnospiraceae* and *Ruminiclostridium*. The propionic acid was positively correlated with *Parasutterella* and *Bifidobacterium*, and negatively correlated with *norank_f_Lachnospiraceae*. The butyric acid was positively correlated with *Parasutterella* and *Bifidobacterium*, and negatively correlated with *Coriobacteriaceae_UCG-002* and *Blautia*. The valeric acid was positively correlated with *norank_o_Mollicutes_RF9*, *Parasutterella*, and *Candidatus_Saccharimonas*. The total SCFAs were positively correlated with *Parasutterella* and *Bifidobacterium* and negatively correlated with *norank_f_Lachnospiraceae* and *Blautia*. For *Torreya grandis* oil exhibited anti-Alzheimer’s disease activity on scopolamine-induced mice, the acetic acid in serum was positively correlated with *Allobaculum*, *Parasutterella*, and *Bifidobacterium* (89). For peony seed oil revealed hypocholesterolemic effect toward high-cholesterol diet-induced hamsters (80), the acetic acid and propionic acid were positively correlated with *unclassifed_f_Erysipelotrichaceae* and *Bifidobacterium*, and negatively correlated with *Ruminiclostridium_9* and *norank_o_Gastranaerophilales*. In addition, the acetic acid was positively correlated with *Anaeroplasm*, and negatively correlated with *Papillibacter*. The butyric acid and valeric acid were positively correlated with *Ruminococcus_2* and *Bifidobacterium*, and the valeric acid was negatively correlated with *norank_o_Gastranaerophilales* and *Butyricimonas*. The total SCFAs were positively correlated with *unclassifed_f_Erysipelotrichaceae* and *Bifidobacterium*, and negatively correlated with *norank_o_Gastranaerophilales* and *Butyricimonas*. For hawthorn seed oil showed hypocholesterolemic effect on high-cholesterol diet-induced hamsters (96), the acetic acid, propionic acid, butyric acid, valeric acid, and total SCFAs in feces were positively correlated with *Ruminococcus_2*, and negatively correlated with eight bacteria (*Ruminiclostridium_9*, *norank_f_Ruminococcaceae*, *Desulfovibrio*, *Butyricimonas*, *Oscillibacter*, etc.). In addition, the acetic acid was negatively correlated with *Lachnospiraceae_NK4A136_group*, *Blautia*, and *Ruminiclostridium_5*. The propionic acid was positively correlated with *Faecalibaculum* and *Anaeroplasm*, and negatively correlated with eight bacteria (*Lachnospiraceae_NK4A136_group*, *Peptococcus*, *norank_o_Gastranaerophilales*, *Ruminiclostridium*, *Ruminococcaceae_NK4A214_group*, etc.). The butyric acid was positively correlated with *Alistipes* and *Faecalibaculum*, and negatively correlated with *norank_o_Gastranaerophilales*, *Ruminococcaceae_NK4A214_group*, *Peptococcus*, *Roseburia*, and *unclassified_f_Christensenellaceae*. The valeric acid was positively correlated with *Faecalibaculum*, and negatively correlated with *norank_o_Gastranaerophilales*, *Ruminococcaceae_NK4A214_group*, *Peptococcus*, *Roseburia*, and *unclassified_f_Christensenellaceae*. The total SCFAs were positively correlated with *Faecalibaculum* and *Anaeroplasm*, and negatively correlated with seven bacteria (*Lachnospiraceae_NK4A136_group*, *norank_o_Gastranaerophilales*, *Ruminiclostridium*, *Peptococcus*, *Blautia*, etc.).

#### 5.3.2 Gut microbiota-derived metabolites and gut microbiota

For camellia oil exhibited gastroprotection on ethanol-induced mice (41), close correlations were found between metabolites and nine bacterial taxa (*Rhodobacter*, *Bosea*, *Bacteroides*, *Dorea*, *Streptococcus*, etc.) in feces. Among them, 40 metabolites (5,6-dimethylbenzimidazole, vincamine, gingerenone, amino acid, etc.) were negatively associated with *Rhodobacter*, *Bosea*, *Bacteroides*, and *Dorea*. Among them, *Bosea* and *Dorea* were two of differential gut microbiota biomarkers for camellia oil. Regarding the anti-diabetes effect of olive oil on NOD/LtJ mice (48), the majority of upregulated serum lipid metabolites were positively correlated with seven types of bacteria (Verrucomicrobia, *Akkermansia*, Cyanobacteria, *Bacteroides*, Gastranaerophilales, etc.), and negatively correlated with *Lachnospira* and *Eubacterium_xylanophilum_group*. Conversely, most decreased serum lipid metabolites were positively correlated with *Lachnospira* and *Eubacterium_xylanophilum_group*, and negatively correlated with *Lachnoclostridium*, *Cyanobacteria*, *Gastranaerophilales*, *Bacteroides*, and *Ruminococcaceae_UCG-005*. Among them, *Akkermansia*, *Bacteroides*, Gastranaerophilales, *Lachnoclostridium*, *Ruminococcaceae_UCG-005*, and *Eubacterium_xylanophilum_group* were differential gut microbiota biomarkers for olive oil. As flaxseed oil exerted anti-atherosclerosis effect against high-fat diet-induced *ApoE*^−/−^ mice (55), the BAs included LCA, ACA, GCA, and TCA were negatively correlated with seven bacteria (*Intestinimonas*, *Bilophila*, *Anaerotruncus*, *Oscillibacter*, *Negativibacillus*, etc.), and alloLCA, isoLCA, 7-ketolca, *β*-UDCA, CDCA, HDCA, and *α*-MCA were positively correlated with these bacteria. For flaxseed oil showed regulation of lipid metabolism activity on high-fat diet-induced mice (51), 12-DHCA, TDCA, and DCA were closely correlated with the most bacterial genera, and the most metabolites were positively correlated with *Coprobacillus*, *Adelercreutzia*, and *Ruminococcus*. Among them, *Ruminococcus* was one of the differential gut microbiota biomarkers for flaxseed oil. For flaxseed oil revealed regulation of lipid metabolism action on Albas cashmere goats (53), significant correlations were observed between blood fatty acid composition and duodenal, ileal, jejunal, cecal, and colonic differential bacteria. Especially, three fatty acids, including c18:3n3, C20:4n6, and c20:5n3, were significantly correlated with differential bacteria (*[Eubacterium]_coprostanoligenes_group*, *Acetitomaculum*, *Anaerofustis*, *Brevibacillus*, *Denitrobacterium*, etc.) in duodenum, jejunum, ileum, cecum, and colon.

For sea buckthorn seed oil exerted hypocholesterolemic effect toward high-cholesterol diet-induced hamsters (69), total neutral sterols, coprostanol, coprostanone, cholesterol, dihydrocholesterol, total acidic sterols, lithocholic acid, deoxycholic acid, chenodeoxycholic acid, and cholic acid were significantly correlated with 19 key genera bacteria (*norank_f_Bacteroidales_S24-7_group*, *Acietatifactor*, *Allobaculum*, *norank_o_Mollicutes_RF9*, *Lactobacillus*, etc.). At the same time, peony seed oil exhibited hypocholesterolemic effect on high-cholesterol diet-induced hamsters (80), these metabolites were closely related to 27 specific genera bacteria (*Allobaculum*, *Anaeroplasm*, *Bifidobacterium*, *Butyricimonas*, *unclassified_f_Ruminococcaceae*, etc.). For hawthorn seed oil showed hypocholesterolemic effect on high-cholesterol diet-induced hamsters (96), these metabolites were obviously correlated with 30 specific genera bacteria (*Faecalibaculum*, *Ruminococcus_2*, *Peptococcus*, *unclassifed_f_Christensenellaceae*, *norank_o_Gastranaerophilales*, etc.). For wild melon seeds oil exhibited hypocholesterolemic effect against high-cholesterol diet-induced hamsters (93), these metabolites were notably correlated with 26 specific genera of bacteria (*Bifidobacterium*, *Ruminococcus_2*, *nonrank_f_Eubacteriaceae*, *Bilophila*, *Blautia*, etc.). On the other hand, for peony seed oil exerted alleviate hyperlipidemia and hyperglycemia action on high-fat diet-induced mice (81), 12 metabolites (adenosine, choline, *β*-asarone, glycitein, betaine, etc.) were notably correlated with 12 bacteria (*Lactobacillus*, *Bifidobacterium*, *Parabacteroides*, *Coprococcus*, *Paraprevotella*, etc.). For *Decaisnea insignis* seed oil displayed hepatoprotection against alcohol-induced mice (82), 25 metabolites (asparagine, L-kynurenine, chlorogenic acid, sarcosine, synephrine, etc.) were obviously correlated with 15 bacteria (*Desulforibrio*, *[Eubacterium]_coprostandigenes_group*, *Akkermansia*, *Ruminoccaceae_UCG_004*, *Streptococcus*, etc.). For sacha inchi oil reflected hypolipidemic effect on high-fat diet-induced rats (84), significant corrections were found between the primary bile acids (CA, GCA, TCDCA, and TCA) in the metabolisms of glycerolipid and glycerophospholipid and the differential bacteria (*Roseburia*, *Turicibacter*, *Butyrivibrio*, *Escherichia*, *Bacteroides*, etc.). For tomato seed oil showed anti-hyperlipidemia activity on high-fat diet-induced mice (94), fecal bile acids (SFA, MUFA, PUFA, and total FA) were closely related to *Lactobacillus*, *Odoribacter*, *Rikenella*, *norank_f__Clostridiales_vadinBB60_group* and/or *[Eubacterium]_fissicatena_group*. Among them, *Lactobacillus* was one of the differential gut microbiota biomarkers for tomato seed oil.

### 5.4 Correlations between altered metabolites and changed biochemical indexes

In terms of SCFAs, for flaxseed oil exerted anti-diabetes action on streptozotocin-nicotinamide-induced rats (55), the acetic acid was negatively correlated with TNF-*α*, IL-1β, IL-6, and IL-17A. Meanwhile, the propionic acid and butyric acid were negatively correlated with IL-1β, IL-6, and IL-17A. For flaxseed oil exhibited anti-atherosclerosis activity in high-fat diet-induced *ApoE*^−/−^ mice (58), the acetic acid and propionic acid were negatively correlated with LPS, TNF-α, IL-1β, and IL-17A in plasma and with TNF-α, IL-1β, and IL-6 in the aorta. The isobutyric acid was positively correlated with IL-10 in the aorta, and negatively correlated with TNF-α and IL-1β in plasma and with TNF-α and IL-6 in the aorta. The isovaleric acid was negatively correlated with TNF-α and IL-1β in plasma and with IL-1β in the aorta. The valeric acid was positively correlated with IL-10 in the aorta, and negatively correlated with LPS, TNF-*α*, IL-1β, and IL-17A in plasma and with TNF-*α*, IL-1*β*, and IL-6 in the aorta.

Regarding other gut microbiota-derived metabolites, olive oil showed anti-diabetes effect on NOD/LtJ mice (48), the increased serum metabolites were positively correlated with islet number, function, and glycemic control, while were negatively correlated with inflammation and cell immunity. For instance, madecassic acid exhibited a positive correlation with islet number but a negative correlation with insulitis, Th1/Th2, MLN_IL-6, AUC area of OGTT and Random blood glucose_15W. Unsaturated fatty acids such as linoleic acid, erucic acid, nervonic acid, and eicosenoic acid were negatively correlated with insulitis and the AUC area of OGTT. Conversely, those decreased lipids, such as oleamide, methyllycaconitine, 5-aminovaleric acid betaine, and ginsenoside f3, were mainly positively correlated with colon_IL-6, insulitis, and AUC area of OGTT. For flaxseed oil exhibited anti-atherosclerosis activity on high-fat diet-induced *ApoE*^−/−^ mice (58), alloLCA and isoLCA were positively correlated with LPS in plasma, and CDCA and HDCA were positively correlated with TNF-*α* in aorta. LCA was negatively correlated with IL-1β and IL-6 in the aorta. 7-ketoLCA was positively correlated with LPS in plasma and TNF-α in the aorta. β-UDCA was positively correlated with IL-1β and IL-17A in plasma and aorta. α-MCA was positively correlated with IL-17A in plasma and with IL-1β and IL-17A in the aorta. ACA was negatively correlated with LPS and TNF-α in plasma. GCA was negatively correlated with LPS, TNF-α, and IL-17A in plasma, and with TNF-α in the aorta. TCA was negatively correlated with LPS, TNF-α, and IL-1β in plasma, and with TNF-α and IL-1β in the aorta. For sacha inchi oil exerted hypolipidemic effect against high-fat diet-induced rats (84), CA was positively correlated with *Cyp7a1* and *Hsd3b7*, and negatively correlated with *Amacr*, *Slc27a5*, *Slc10a1*, and *FXR*. GCA was negatively correlated with *Slc27a5*, *Slc10a1*, and *Abcc2*. TCDCA was negatively correlated with *Amacr*, *Slc27a5*, and *Slc10a1*. TCA was negatively correlated with *Slc27a5* and *Slc10a1*. Meanwhile, lipids (TAG (16:0/16:0/22:4), DG (18:1/20:0), LysoPC (18:2), PC (18:0/20:4), PE (18,1/22:4), etc.) involved in the metabolisms of glycerolipid and glycerophospholipid were significantly correlated with differential genes (*Pemt*, *Crls1*, *Lipc*, *Acadm*, *Scd2*, etc.).

## 6 Conclusion and prospects

**Figure 5:**
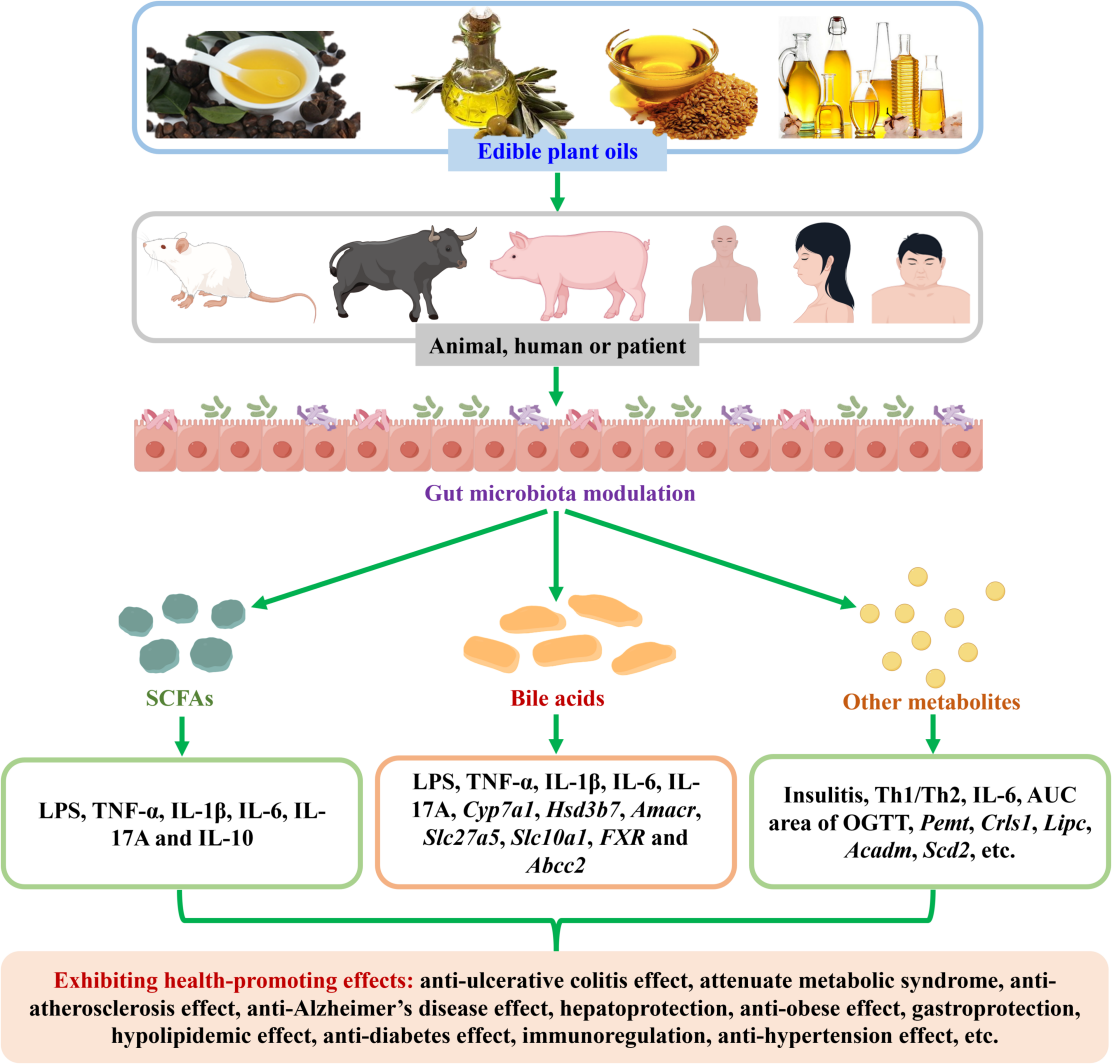
The speculation of “edible plant oils→gut microbiota→metabolites→biochemical indexes→health-promoting effects”.

Many edible plant oils (especially camellia oil, olive oil, and flaxseed oil) could modulate gut microbiota during their health-promoting effects. Moreover, the modulated gut microbiota was significantly correlated with the changed biochemical indexes. Furthermore, the altered metabolites by edible plant oils were obviously correlated with the modulated gut microbiota and changed biochemical indexes. Overall, it could be speculated that edible plant oils modulate gut microbiota to alter SCFAs and other gut microbiota-derived metabolites, thereby changing biochemical indexes to exhibit health-promoting effects (Figure 5). This review provides theoretical basis for understanding and future research in health-promoting effects of edible plant oils from the perspective of gut microbiota modulation.

There are still some questions that remain to be solved: (i) the interactions between edible plant oils and gut microbiota have been rarely studied, and the key bioactive nutrients for edible plant oils in modulating gut microbiota during their health-promoting effects have not been well-ascertained; (ii) which gut microbiota and metabolites are exactly important for edible plant oils in exerting health-promoting effects, and how the screened differential gut microbiota biomarkers along with the altered metabolites mediate the health-promoting effects of edible plant oils, are unknown; (iii) the mechanisms of edible plant oils in modulating gut microbiota, and gut microbiota altering metabolites as well as metabolites changed biochemical indexes have not been investigated; (iv) cohort or clinical studies and the related specific mechanisms concerning gut microbiota modulation of edible plant oils have been rarely uncovered, and the relevant existing researches were absence of longitudinal data.
